# Impaired Aire-dependent IFN signaling in the thymus precedes the protective autoantibodies to IFNα

**DOI:** 10.1084/jem.20241403

**Published:** 2025-04-30

**Authors:** Artur Stoljar, Maksym Zarodniuk, Rudolf Bichele, Elise Helene Armulik, Uku Haljasorg, Romain Humeau, Marine Besnard, Liis Haljasmägi, Liina Tserel, Merili Peltser, Ahto Salumets, Eliisa Kekäläinen, Kai Kisand, Carole Guillonneau, Martti Laan, Pärt Peterson

**Affiliations:** 1 Institute of Biomedicine and Translational Medicine, University of Tartu, Tartu, Estonia; 2 Translational Immunology Research Program, Faculty of Medicine, University of Helsinki, Helsinki, Finland; 3 Nantes University, INSERM, Center for Research in Transplantation and Translational Immunology, UMR 1064, CNRS, Nantes, France

## Abstract

Recent studies have highlighted the role of the thymus in maintaining immune tolerance to type 1 interferons (T1 IFNs). Individuals with thymic abnormalities, such as autoimmune regulator (AIRE) gene mutations, frequently develop neutralizing autoantibodies to interferon-alpha (IFNα). Unlike mice, Aire-deficient rats develop robust autoantibodies to IFNα. Using this rat model, we show that Aire regulates the thymic expression of interferon-stimulated genes (ISGs), which occurs before developing anti-IFNα autoantibodies. In the periphery, we observed a widespread downregulation of ISGs across immune cells and reduced activation of natural killer (NK) cells. Furthermore, the presence of anti-IFNα autoantibodies correlated with reduced peripheral tissue inflammation, suggesting their role in dampening T1 IFN signaling and minimizing tissue infiltration. Our findings reveal that Aire-mediated regulation of thymic T1 IFN signaling is linked to the production of protective anti-IFNα autoantibodies, which inversely correlate with autoimmune pathology in peripheral tissues.

## Teaser

AIRE regulates thymic interferon-stimulated genes, preceding anti-IFNα autoantibody production in Aire-deficient rats. These autoantibodies reduce T1 IFN signaling, dampen immune activation, and correlate with lower tissue inflammation, uncovering a protective mechanism that inversely associates with autoimmune pathology.

## Graphical Abstract

**Figure:**
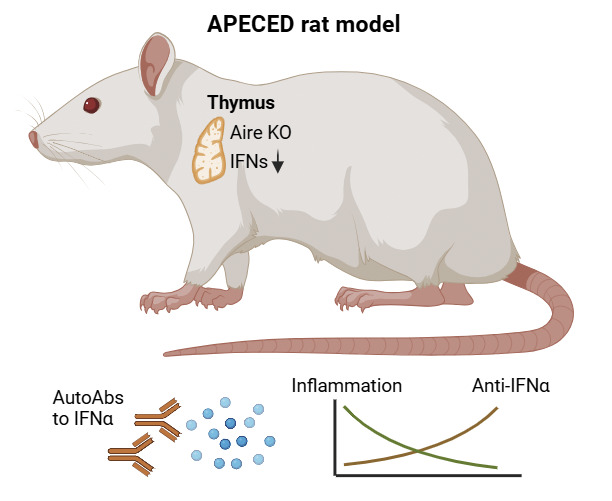

## Introduction

Type 1 interferons (T1 IFNs) are cytokines primarily stimulated by pathogens to trigger immune responses against viruses. Produced by most cell types and acting at extremely low concentrations, they initiate a signaling cascade that results in the upregulation of hundreds of interferon-stimulated genes (ISGs). These genes encode proteins involved in diverse cellular processes, such as inhibiting viral replication, and modulating innate and adaptive immunity.

However, beyond being activated by viral DNA or RNA, they have broader immunomodulatory functions in host defense and immune homeostasis (González-Navajas et al., 2012; Gough et al., 2012; Ivashkiv and Donlin, 2014). Recent studies suggest that a constitutive low-level expression of T1 IFNs in the thymus may play specific roles in shaping T-cell development and that the thymus is necessary to maintain T-cell tolerance to interferons (IFN) (Ashby et al., 2024; Martinez and Hogquist, 2023).

Critical in maintaining central tolerance, the autoimmune regulator (AIRE) gene is mainly expressed in medullary thymic epithelial cells (mTECs), where it promotes the expression of tissue-restricted antigen (TRA) genes that are recognized by developing thymocytes (Anderson et al., 2002; Mathis and Benoist, 2009; Peterson et al., 2008). The lack of self-antigen expression in the thymus of Aire-deficient mice results in defective negative selection of the T cell repertoire and the impaired development of regulatory T cells (Tregs), thereby contributing to autoimmunity (Anderson et al., 2005; Liston et al., 2003).

Patients with the mutations in the AIRE gene develop a multicomponent disease known as autoimmune polyendocrinopathy–candidiasis–ectodermal dystrophy (APECED) that is characterized by several autoimmune diseases, in particular those targeting the endocrine organs (Kisand and Peterson, 2015; Meager et al., 2006). Anti-IFNα autoantibodies are present in up to 95–100% of APECED patients and can be used as diagnostic markers for the disease (Kisand et al., 2008; Meager et al., 2006; Wolff et al., 2007). Commonly, they are neutralizing at nanomolar concentrations, active in vivo, and their neutralizing efficiency in APECED patients has an inverse correlation with the incidence of Type 1 diabetes (T1D), suggesting that these naturally occurring autoantibodies can mitigate the autoimmunity seen in patients (Fishman et al., 2017; Meyer et al., 2016). In addition to APECED, autoantibodies targeting cytokines are not unique to APECED. They are present in other inborn errors, potentially affecting thymus-associated central tolerance such as IPEX, caused by defects in the *FOXP3* gene; hypomorphic *RAG1/RAG2* gene mutations; immunodeficiencies owing to *NFKB2*, *NIK*, *RELB*, *CTLA4*, *IKZF2*, and *CD40L* defects; and in thymomas, which have a disorganized thymus with decreased expression of MHC class II and AIRE (Cheng and Holland, 2024; Hetemäki et al., 2021; Le Voyer et al., 2023; Oftedal et al., 2024), highlighting the role of the thymus in maintaining tolerance to IFNs.

Despite being the most prominent marker of APECED and being present in several other diseases with defects in thymic tolerance, the etiology and functional impact of T1 IFN autoantibodies in disease pathogenesis have remained enigmatic. In part, this is because of the limitations of Aire-deficient mouse models. While Aire-deficient mouse models have been instrumental in deciphering the mechanism of thymic tolerance (Anderson et al., 2002; Kuroda et al., 2005; Liston et al., 2004) and showing decreased expression of a broad repertoire of self-antigens and development of autoantibodies to tissue-specific antigens, they display a conspicuously mild disease and lack anti-IFNα autoantibodies. By contrast, the recently characterized Aire-deficient rat model develops autoantibodies to IFNα, similar to human patients (Besnard et al., 2022; Ossart et al., 2018). Aire-deficient rats have decreased expression of TRAs in the thymus, autoantibodies to multiple other targets, and lymphocytic infiltrations in several tissues, with overall pathology closer to APECED patients than that shown by the Aire-deficient mice.

Therefore, by using the Aire-rat model, we explored the role of AIRE in maintaining tonic T1 IFN signaling in the thymus and investigated the effect of anti-IFNα autoantibodies on peripheral immune infiltrations, and found a surprising correlation between these cytokine autoantibodies and the inflammatory processes in affected tissues, providing new insights into the relationship between thymic tolerance, cytokine autoantibodies, and autoimmune pathogenesis.

## Results

### All Aire-deficient rats develop neutralizing anti-IFNα autoantibodies in immune tissues

**Figure 1.**
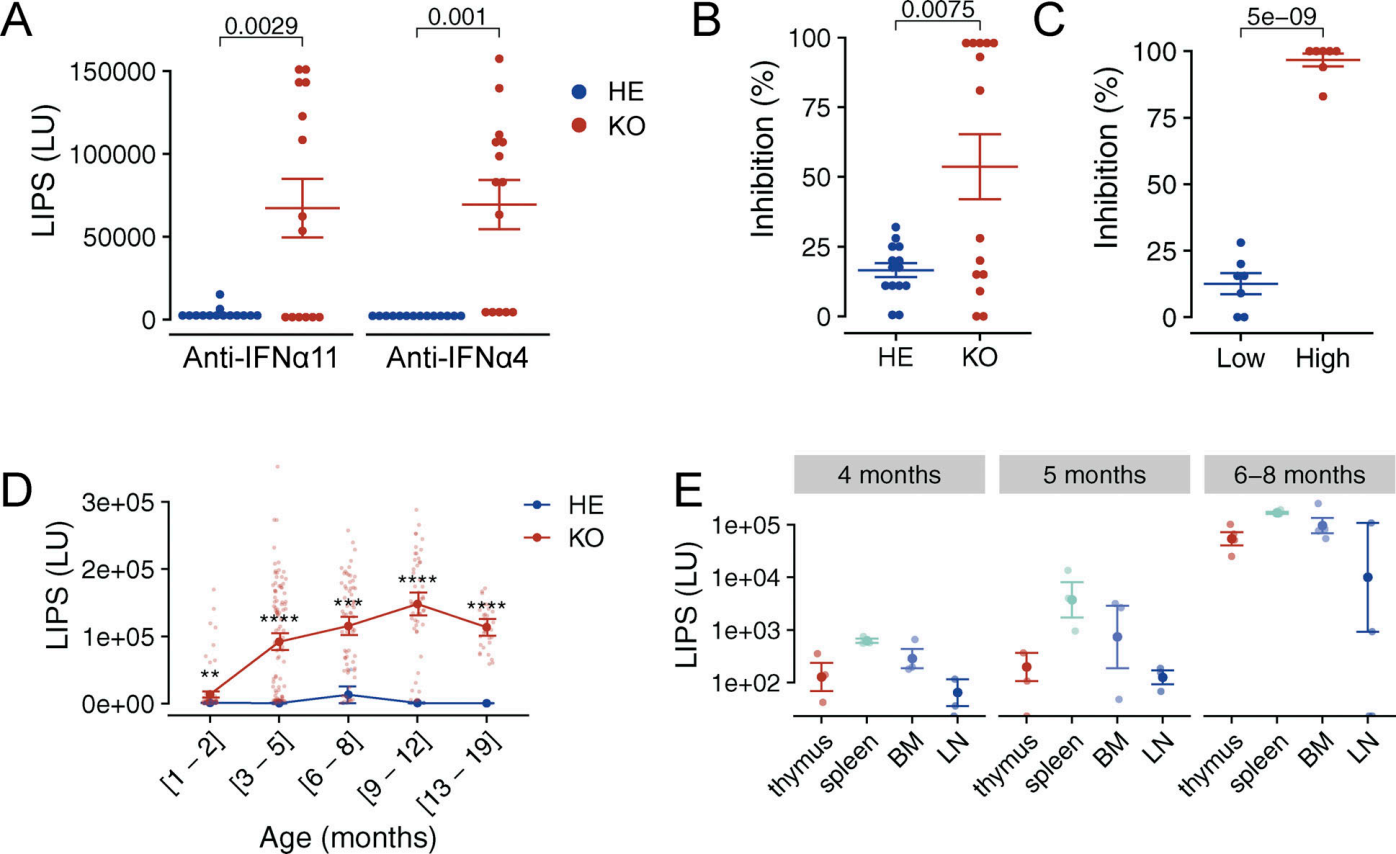
**Autoantibodies to IFNα in Aire-deficient rats. (A)** Autoantibodies to IFNα11 and IFNα4 in the blood plasma of 4–5-mo-old Aire-deficient (KO) and control (HE) rats; each dot represents a luminescence value unit (LU) for one individual animal (*n* = 14 for both KO and HE rats). **(B)** Cell-based assay of IFNα neutralization with Aire-deficient rat plasma (*n* = 14 for both KO and HE rats). **(C)** Neutralization effect of plasma samples containing low and high levels (below and above median) of autoantibodies (*n* = 14 for both KO and HE rats). **(D)** Autoantibody levels to IFNα11 at different ages of the animals (*n* = 62 for KO and *n* = 30 for HE rats). **(E)** Autoantibodies to IFNα11 measured in the supernatant of cells cultured from thymi, spleens, bone marrow (BM), and lymph nodes (LN) of Aire-deficient rats at different time points (*n* = 4). Throughout the figure, symbols indicate individual rats measured in batches of 5–20 rats per experiment. Horizontal lines with whiskers indicate mean values with SEM. All reported P values are based on *t* tests. P values are adjusted for multiple comparisons using the Holm–Bonferroni method. Statistical significance is indicated as follows: ****P < 1e-4, ***P < 0.001, **P < 0.01.

**Figure S1.**
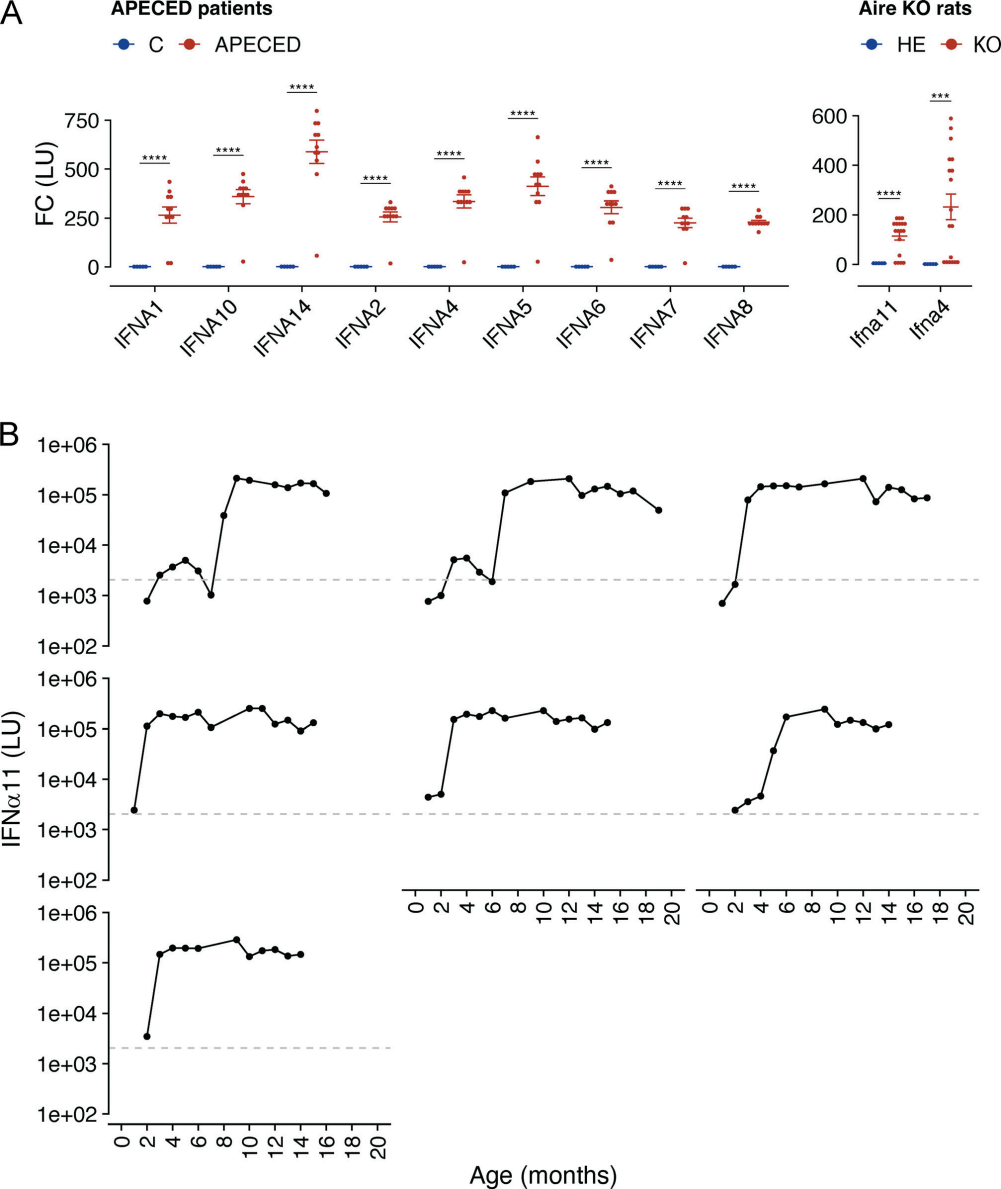
**Anti-IFNα autoantibodies in APECED patients and time course of the development of anti-IFNα autoantibodies in individual Aire-deficient rats. (A)** Titers for antibodies against human IFNα subtypes in the blood plasma of APECED patients and control individuals (left) and anti-IFNα11 and IFNα4 subtypes in Aire-deficient (KO) and control (HE) rats (right) as measured by LIPS assay (APECED *n* = 11, controls *n* = 5; KO *n* = 18, HE *n* = 5; FC, fold change). P values are reported based on *t* tests. P values are adjusted for multiple comparisons using the Holm–Bonferroni method. Statistical significance is indicated as follows: ****P < 1e-4, ***P < 0.001. **(B)** Time course for the development of IFNα11 antibodies in the blood plasma of seven individual Aire-deficient rats as measured by LIPS. The cut-off line indicates the mean of antibody titers of control rats plus two standard deviations. In A, symbols indicate individual humans or animals and are measured in batches from 5 to 20 samples per experiment. Horizontal lines with whiskers indicate mean values with SEM.

Like APECED patients, Aire-deficient rats develop high levels of anti-IFNα autoantibodies, although their developmental dynamics and neutralizing capacity remain to be elucidated. To quantify and characterize the presence of these autoantibodies, we measured anti-IFNα4 and anti-IFNα11 autoantibody levels in 4–5-mo-old Aire-deficient animals using a luciferase-based LIPS assay. Approximately 50% of Aire-deficient rats at this age, and none of the heterozygous rats, had detectable levels of autoantibodies (Fig. 1 A). The luciferase units representing antibody levels ranged 150–600-fold higher values in Aire-deficient animals than in controls, comparable with those observed in APECED patients (Fig. S1 A) (Meyer et al., 2016). Notably, the anti-IFNα autoantibodies exhibited potent neutralizing capacities, as demonstrated by the ability of plasma samples from Aire-deficient rats to inhibit mouse IFNα-induced luciferase expression in the RAW-Lucia ISG reporter cell line (Fig. 1 B). The animals with high anti-IFNα autoantibody levels achieved almost 100% neutralization capacity, indicating that antibody levels were strongly associated with neutralizing efficiency (Fig. 1 C).

To assess the developmental progression of the anti-IFNα autoantibodies, we examined their levels in Aire-deficient and control rats at ages ranging from 1 to 19 mo. In Aire-knockout animals, anti-IFNα autoantibodies became detectable at 2 mo of age, with titers peaking between 7 mo and 1 year, after which there was no further increase (Fig. 1 D). However, the timing for the autoantibody appearance varied after the second month, indicating interindividual diversity, as we found in a longitudinal autoantibody analysis of seven knockout animals (Fig. S1 B). Nevertheless, at the 1-year mark, all rats with a homozygous (but none of the heterozygous) Aire mutation had developed high levels of anti-IFNα autoantibodies.

Next, we aimed to determine whether the autoantibody generation in Aire-deficient rats was confined to specific immune tissues. We measured anti-IFNα autoantibody levels in the supernatants of ex vivo cell cultures obtained from thymi, spleens, bone marrow, and lymph nodes of Aire-deficient rats. To confirm de novo autoantibody production, we cultured tissue-extracted lymphocytes from animals of different ages for 2 wk with periodic culture medium changes. A signal of anti-IFNα autoantibodies emerged in tissues collected from 5-mo-old animals but was substantially higher in Aire-deficient rats aged over 6 mo (Fig. 1 E). Although the highest autoantibody levels were present in splenocyte cultures, high-level IFNα autoantibodies were produced by cells from all immune tissues, including the thymus. This result indicated a widespread distribution of autoantibody-producing B cells throughout all immune organs rather than confined to a specific site.

Together, these results show the development of highly neutralizing anti-IFNα autoantibodies in all Aire-deficient rats as they age, with their production evident across multiple immune tissues. The consistent presence of these autoantibodies at elevated levels and their potent neutralizing potential underscore the suitability and robustness of the Aire-deficient rat as an experimental model to study the anti-IFNα response in APECED pathology.

### Reduced numbers of thymic Tregs in Aire-deficient rats

To gain insight into the thymic changes in the Aire-deficient rat model, we investigated the proportions of thymocytes and thymic epithelial cells and examined thymic architecture by flow cytometry and immune fluorescence. To exclude any confounding effect from neutralizing anti-IFNα autoantibodies, we used young Aire-knockout rats aged 4–6 wk, in which we verified the absence of detectable autoantibodies to IFNα prior to this analysis.

**Figure 2.**
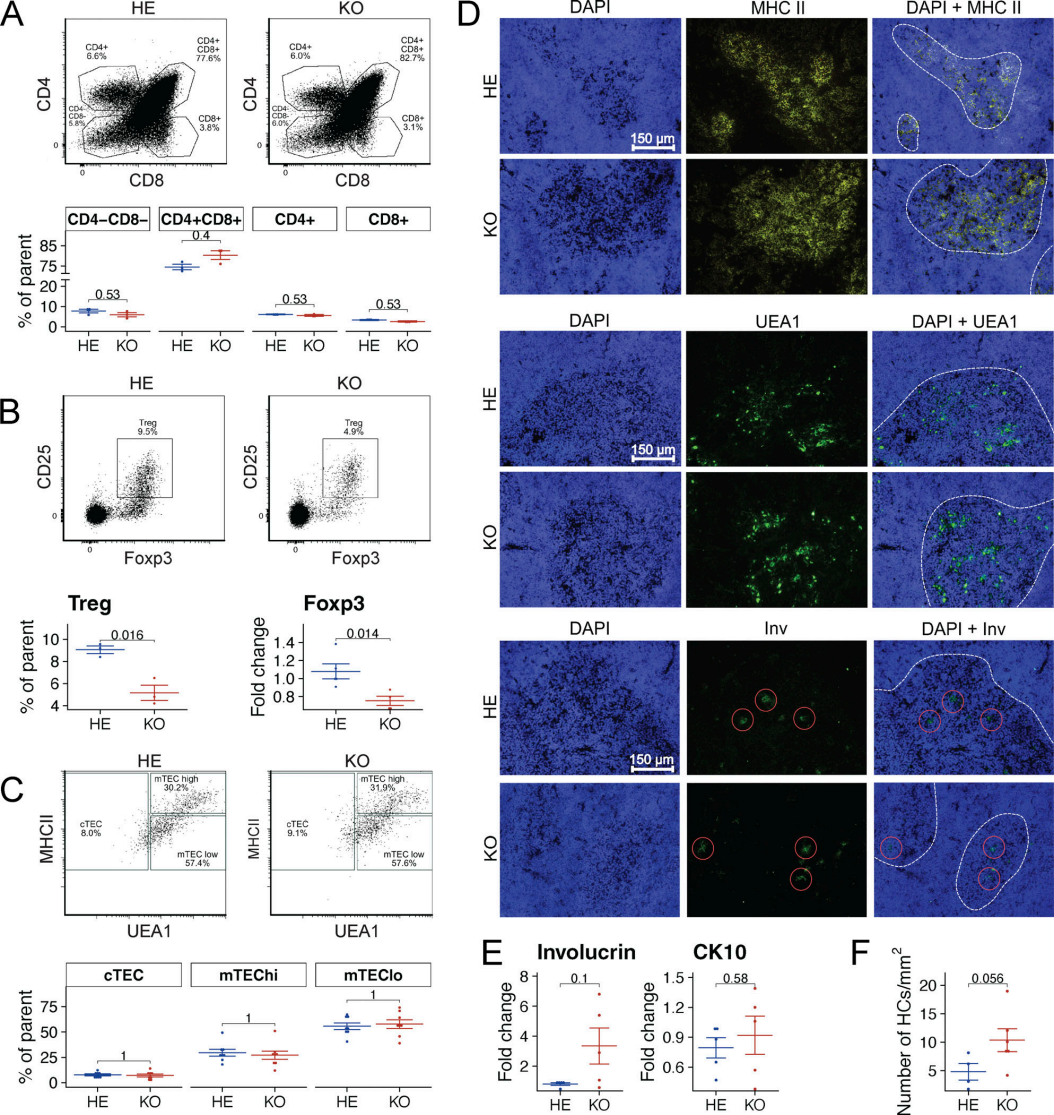
**Aire impact on thymocyte and mTEC maturation. (A)** The percentages of four main thymocyte populations in the thymi of Aire-deficient (KO) and control (HE) rats as determined by flow cytometry. Representative flow cytometry plots (above) and percentages of DN, DP, and SP cell populations in Aire-deficient and control rats (below, *n* = 3 for both KO and HE animals, combined from two independent experiments). **(B)** Representative flow cytometry plots of thymic Tregs (above), the percentages of Treg population in the thymi determined by flow cytometry (left below, *n* = 3 for both KO and HE rats, combined from two independent experiments) and relative *Foxp3* mRNA expression in the thymus determined by qPCR (right below, *n* = 5 for both KO and HE animals, combined from two independent experiments). **(C)** Representative flow cytometry plots (above) and the percentages of three TEC subpopulations in the thymi as determined by flow cytometry (*n* = 8 for both KO and HE rats, combined from two independent experiments). **(D)** Thymic sections were stained for MHC II, UEA1, and Involucrin; dashed line represents the medulla–cortex border; red circle represents Involucrin^+^ Hassall’s corpuscles. Shown are representative samples from three independent experiments, scale bar = 150 μm. **(E)** Relative Involucrin (*Inv*) and Cytokeratin 10 (*CK10*) expression in the thymus as determined by qPCR (*n* = 5 for both KO and HE rats, combined from two independent experiments). **(F)** Average number of Involucrin^+^ Hassal’s corpuscles per 1 mm^2^ medulla (*n* = 5 for KO and *n* = 4 for HE animals, combined from three independent experiments). Throughout the figure, symbols indicate individual rats, horizontal lines with whiskers indicate mean values with SEM. All reported P values are based on *t* tests. P values are adjusted for multiple comparisons using the Holm–Bonferroni method.

**Figure S2.**
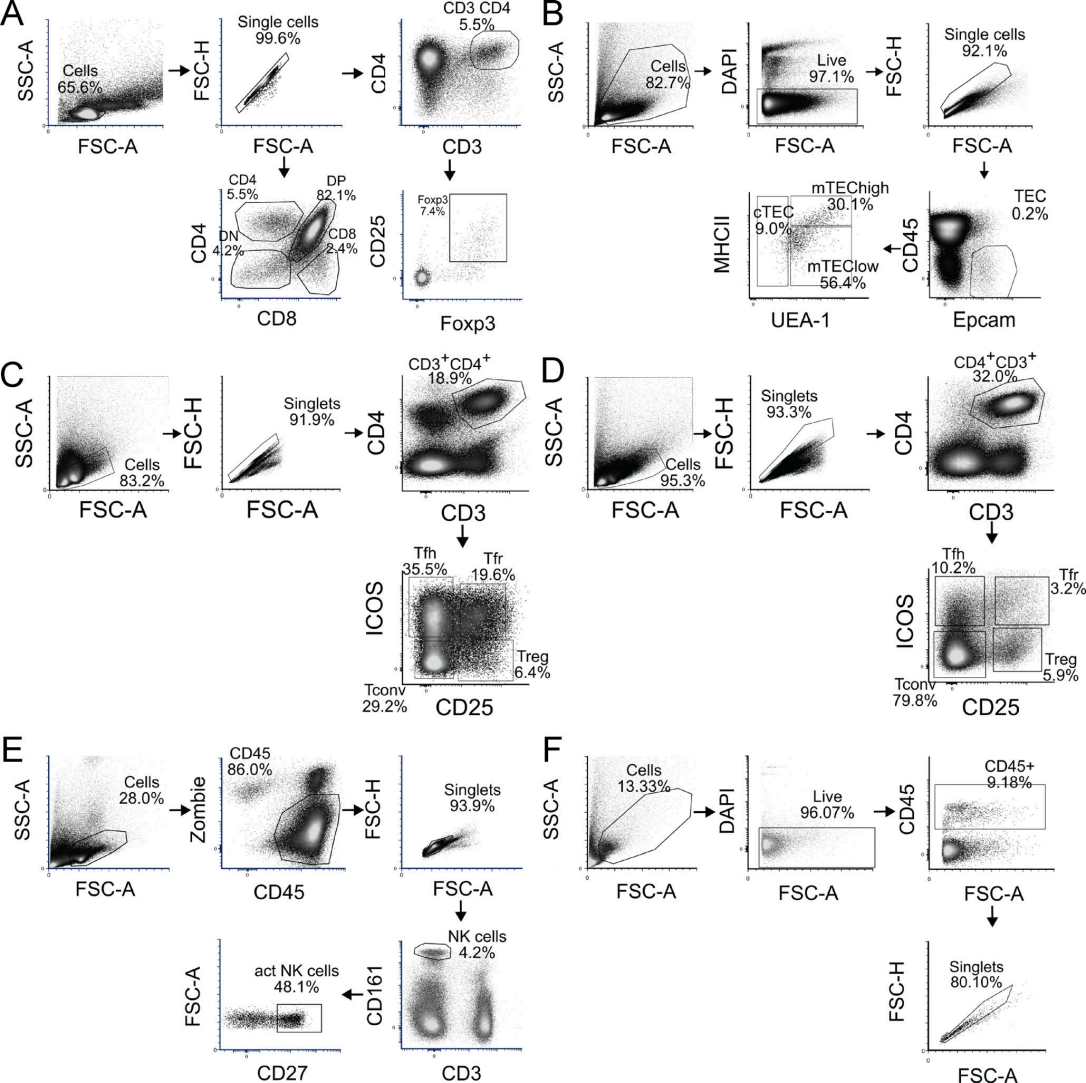
**Gating strategies for flow cytometry. (A)** Gating strategy for DN, DP, CD8^+^, CD4^+^ (SP) T cells, and Foxp3^+^ cells. **(B)** Gating strategy for mTEC^hi^, mTEC^lo^, and cTEC populations. **(C)** Gating strategy for splenic Tfh, Tfr, Tconv, and Tregs. **(D)** Gating strategy for lymph node Tfh, Tfr, Tconv, and Tregs. **(E)** Gating strategy for activated natural killer (NK) cells. **(F)** Gating strategy for CD45^+^ cells in the salivary glands.

The Aire-deficient rats exhibited overall normal thymocyte development with comparable frequencies of double negative (DN), double positive (DP), and single positive (SP) CD4^+^ and CD8^+^ thymocyte populations to those in heterozygous animals (Fig. 2 A and Fig. S2). Importantly, the Aire-deficient rats displayed diminished numbers of thymic Tregs and reduced expression of thymic Foxp3 (Fig. 2 B), confirming that dysregulated thymic Treg development is a key feature of Aire-deficiency.

Analysis of mTEC differentiation, however, showed no discrepancies in the frequencies of the main TEC populations (Fig. 2 C). Moreover, we observed no significant alterations in the thymic expression of MHC II and mTEC marker UEA-1 (Fig. 2 D). The markers for terminally differentiated epithelium involucrin (Fig. 2 D), cytokeratin CK10 (Fig. 2 E), and the number of Hassall’s corpuscles (Fig. 2 F) were not significantly changed, although there was a trend of increased number of terminal mTECs in the knockout animals. Thus, whilst these findings confirm the role of Aire in regulating Treg differentiation, we could not find major changes in mTEC maturation seen in Aire-deficient mice.

### Three mTEC subsets expressed Aire in the rat thymus

**Figure 3.**
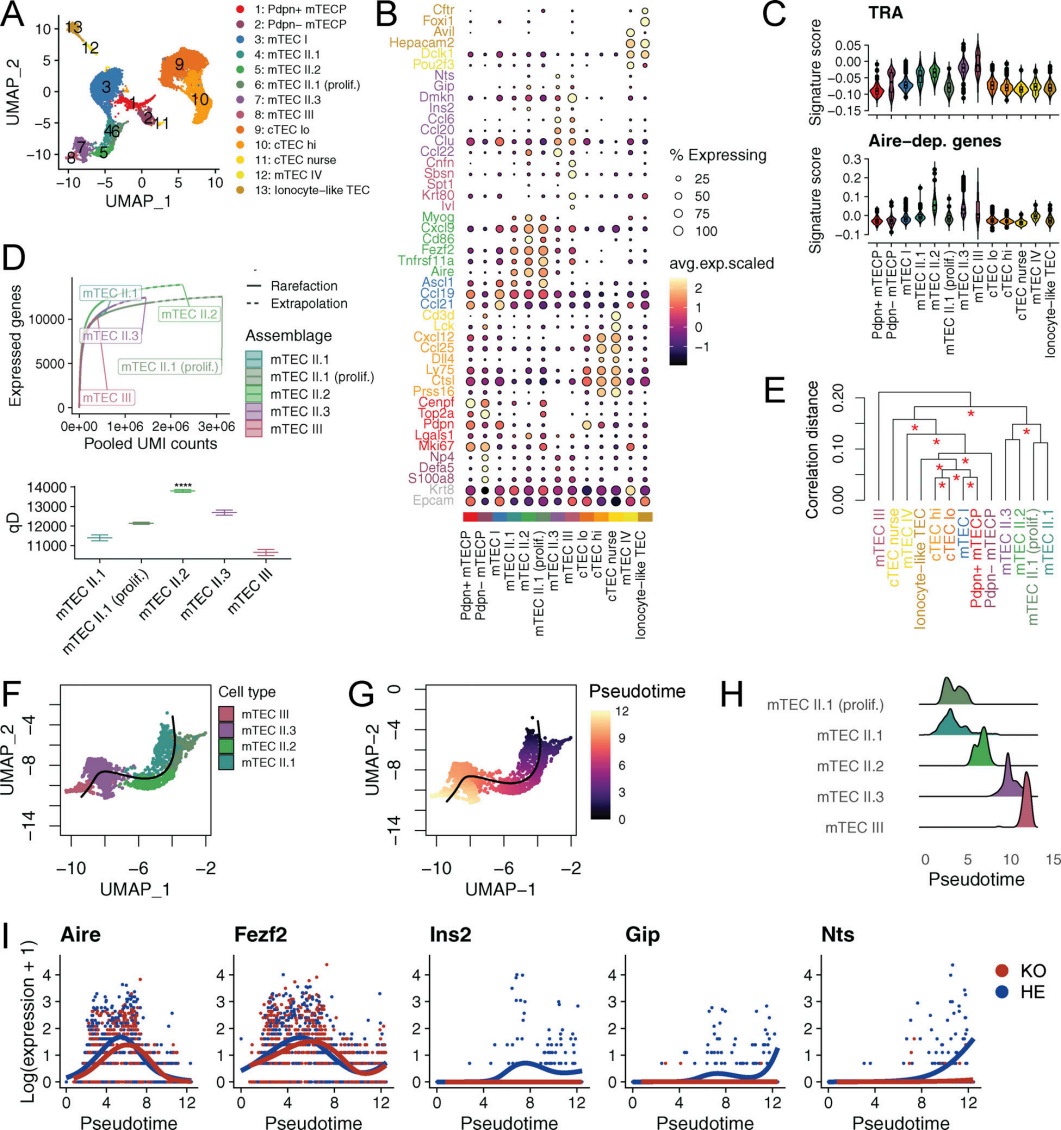
**scRNA-seq reveals mTEC heterogeneity in rat thymus. (A)** Uniform Manifold Approximation and Projection (UMAP) embedding of single-cell transcriptomic data from Aire-deficient and control TECs. **(B)** Dot plot for expression of marker genes. Color represents scaled normalized mean expression of marker genes in each TEC population, and size indicates the fraction of cells expressing marker genes. **(C)** Module scores for TRA and Aire-dependent (Aire-dep) gene sets computed using Seurat’s AddModuleScore confirm the identity of mTEC II subpopulations. Aire-dependent genes were defined by analyzing bulk transcriptome data from purified Aire-deficient and control mTEC^hi^ cells. Tissue-restricted genes were inferred as outlined in Materials and methods. **(D)** Upper panel: number of genes expressed by mTEC subpopulations as a function of UMIs considered. Rarefaction curves were interpolated and extrapolated using the iNEXT R package. Lower panel: transcriptomic diversity of TEC populations, quantified as a Hill number (qD) of order 0 (****P < 1e-4, P values are calculated using Wald-type Z-test based on confidence interval-derived standard errors with mTEC II.2 as a reference group). **(E)** Hierarchical clustering of TEC population based on gene expression correlation distance supports the functional proximity of mTEC II subpopulations. Red asterisks at each node indicate distinct clusters (P < 0.05) based on bootstrap resampling using pvclust R package. **(F and G)** Pseudotime trajectory curve describing differentiation of the mTEC lineage overlaid on UMAP showing mTEC subpopulations (F) and pseudotemporal ordering (G). **(H)** Distribution density plots of mTEC subpopulations ordered along pseudotime showing the successive stages of differentiation. **(I)** Log-normalized expression of selected genes in Aire-deficient and control rats showing diminished expression of Aire-regulated genes in post-Aire mTEC populations. Throughout the figure, *n* = 4 for KO and *n* = 3 for HE animals, and the data are obtained from a single experiment.

**Figure S3.**
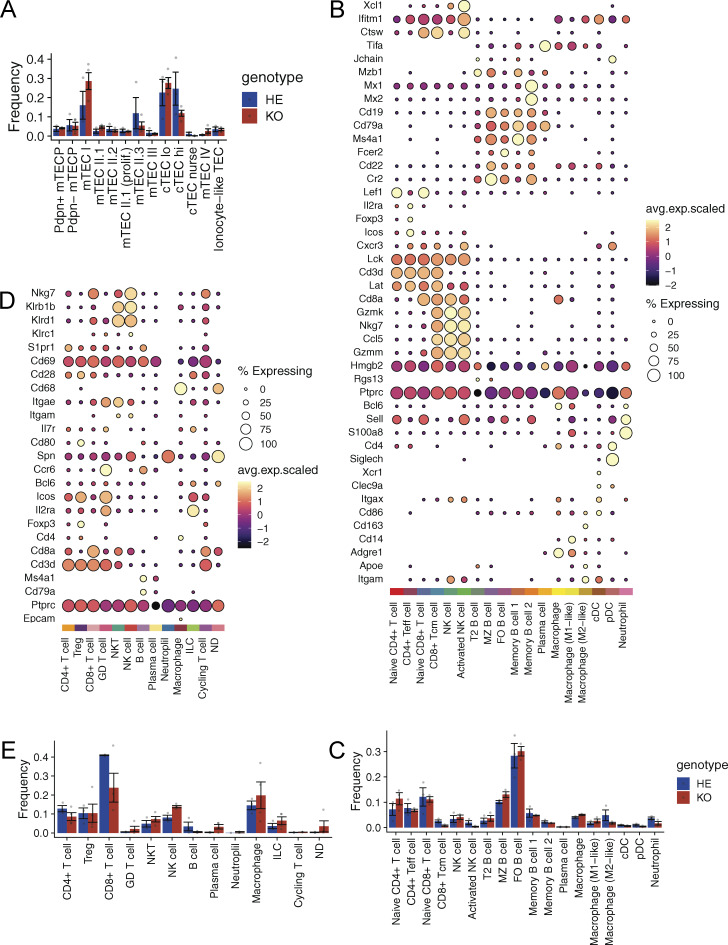
**Gene expression markers and compositional differences in scRNA-seq datasets**. **(A, C, and E)** Frequency of each population in scRNA-seq data from thymic epithelial cells (A), splenocytes (C), and salivary gland immune infiltrates (E). Each dot represents one animal. Data are presented as mean value with SEM and are obtained from one experiment with four Aire-deficient (KO) and three control (HE) rats per group for thymic epithelial cell and salivary gland immune infiltrate analysis and with three Aire-deficient (KO) and three control (HE) rats per group for splenocyte cell analysis. **(B and D)** Gene expression markers used for annotation of the splenocyte (B) and salivary gland (D) data. GD, gamma-delta; ILC, innate lymphoid cell; NK, natural killer; Teff, effector T cell; MZ, marginal zone; cDC, conventional dendritic cell, pDC, plasmacytoid dendritic cell.

To assess the impact of Aire deficiency on thymic stromal cell subpopulations in rats, we employed a single-cell RNA sequencing (scRNA-seq) approach on the purified TEC compartment. We used flow cytometry to sort CD45^−^, EpCAM^+^ cells from 6-wk-old Aire-knockout and control rats to analyze their stromal cell populations using the 10x Genomics platform. scRNA-seq data were clustered and manually annotated using previously identified marker genes in mouse and human TECs (Fig. 3, A and B). Three clusters corresponded to classically characterized cortical thymic epithelial cell (cTEC) populations: cTEC^lo^and cTEC^hi^, which clustered together, and the thymic nurse cells. Two populations clustered between mTECs and cTECs subsets but showed high expression of the *Ki67* marker and other proliferation-related genes, likely represented by *Pdpn*^−^ and *Pdpn*^+^ TEC progenitor populations, respectively. The largest cluster among the previously characterized mTECs was the CCL21^+^ mTEC I population. We additionally identified two smaller clusters corresponding to tuft-like mTEC IV and ionocyte-like mTECs, previously characterized in humans and mice (Michelson et al., 2022). We did not find significant compositional changes in TEC subpopulations, although there was a trend toward increased mTEC I frequency (Fig. S3 A) in Aire-deficient rats.

In the analysis of the Aire-positive mTEC-lineage, we were able to distinguish several subclusters that represented successive stages of differentiation, which we termed proliferating mTEC II.1, mTEC II.1, mTEC II.2, mTEC II.3, and mTEC III, respectively. Of these, the expression of Aire characterized proliferating mTEC II.1, mTEC II.1, and mTEC II.2. Proliferating mTEC II.1 cluster was different from the others by its higher expression of *Ki67* and other cell division-related genes, suggesting it to be a transit-amplifying population for this lineage. mTEC II.1 showed low expression of co-stimulatory molecule CD86 (Fig. 3 B) and moderate expression of TRAs and Aire-dependent genes (Fig. 3 C). In contrast, mTEC II.2 exhibited high expression of *Aire*, *Fezf2*, *Cd86*, and Aire-dependent genes. This cell population expressed the highest number of Aire-dependent genes (Fig. 3 C) and genes overall (Fig. 3 D), aligning closely with the mTEC^hi^ population characterized by flow cytometry. The marker genes *Ccl22* and *Sbsn* defined the mTEC II.3 and mTEC III stages (Bornstein et al., 2018; Michelson et al., 2022).

Interestingly, the highest number of TRAs were expressed by the remaining two populations, mTEC II.3 and mTEC III (Fig. 3 C). The mTEC II.3 exhibited low expression of *Aire* and *Cd86* but high expression of *Ccl20*, *Ccl22*, and TRAs. The mTEC III showed expression of genes typical of keratinocyte differentiation, such as *Ivl*, *Krt80*, *Sbsn*, and *Cnfn*, which likely correspond to the post-Aire mTEC population. Hierarchical clustering of TEC populations based on gene expression correlation distance supported the functional proximity of mTEC II subpopulations because of their relatedness to Aire and Aire-dependent gene expression and distinct hierarchical clustering of mTEC III, which likely differed because of the keratinocyte-like gene expression (Fig. 3 E).

We next established the pseudotemporal relation between the five mTEC lineage-related subpopulations (Fig. 3 F), which showed a sequential order of differentiation as mTEC II.1 proliferating > mTEC II.1 > mTEC II.2 > mTEC II.3 > mTEC III (Fig. 3, G and H). *Aire* and *Fezf2* expression peaked in mTEC II.2 population along this trajectory and were nearly absent in mTEC III keratinocyte-like cells. Notably, post-*Aire* populations showed a loss of the expression of Aire-regulated genes *Ins2*, *Gip*, and *Nts*, highlighting their association with the *Aire* expression in the terminally differentiated mTECs (Fig. 3 I).

Together, our scRNA-seq analysis showed three thymic epithelial cell subsets expressing Aire in the rat thymus (proliferating mTEC II.1, mTEC II.1, and mTEC II.2), of which mTEC II.2 had the highest expression of *Aire*, *Cd86*, Aire-dependent genes, and the number of all expressed genes.

### Decreased expression of TRAs and ISGs in Aire-deficient mTECs

**Figure 4.**
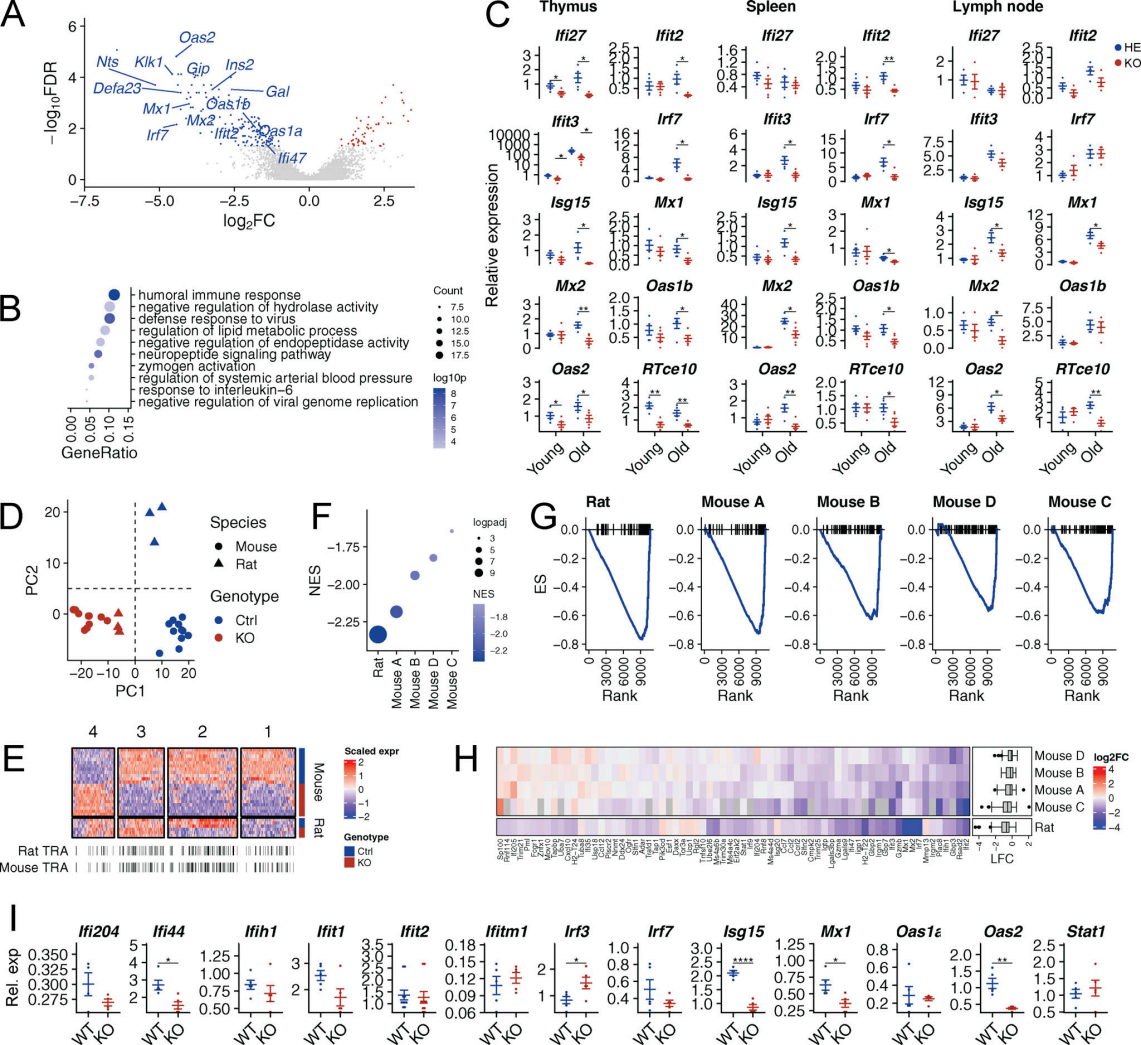
**Comparative analysis of Aire-regulated genes in rat and mouse. (A)** A volcano plot comparing mTEC gene expression in Aire-deficient and control animals. Red and blue dots indicate up- and down-regulated genes, respectively. Data were obtained from a single experiment with 4 Aire-deficient (KO) and 3 control (HE) rats per group. **(B)** Gene ontology (GO) enrichment analysis of Aire-regulated (i.e., downregulated) genes in rats shows enrichment of pathways related to defense response. Only 10 most statistically significant GO terms are shown. **(C)** Gene expression levels as measured by qPCR of ISGs in whole thymi, spleens, and lymph nodes of 1–1.5-mo-old (young) and >7-mo-old (old) Aire-deficient (KO) and control (HE) rats (*n* = 5–6, combined from two or more experiments). **(D)** Principal component analysis of Aire-regulated genes in rat and mouse shows a species-specific effect of Aire. **(E)** Heatmap showing normalized scaled gene expression of Aire-regulated genes in rat and mouse. The genes were clustered into four groups using K-means clustering. Mouse and rat TRA genes are indicated on the right. **(F)** GSEA comparing the degree of ISG downregulation in Aire-deficient rats and mice. Color scale shows the normalized enrichment score (NES) and dot size represents -log10 of the associated P value. **(G)** Running enrichment score (ES) for ISGs in each rat and mouse dataset. **(H)** Heatmap showing log fold changes for ISG genes in rat and mouse datasets. **(I)** Gene expression levels as measured by qPCR of ISGs in whole thymi of 1.5-mo-old Aire-deficient (KO) and WT mice (*P < 0.05, *n* = 5 for both KO and WT, combined from two or more experiments). In C and I symbols indicate individual animals, horizontal lines with whiskers indicate mean values with SEM. All reported P values are based on *t* tests. Statistical significance is indicated as follows: ****P < 1e-4, **P < 0.01, *P < 0.05, and ns (not significant) for P ≥ 0.05.

The key role of Aire in promoting the expression of TRAs in mTECs has been demonstrated by several studies in Aire-deficient mice (Anderson et al., 2002; Derbinski et al., 2005; Kont et al., 2008) and was shown on the whole thymus level in Aire-deficient rats (Ossart et al., 2018). By transcriptome profiling, we confirmed the decreased expression of TRAs in the FACS-sorted CD45^−^, EpCAM^+^, and MHC II^hi^ population from 4- to 6-wk-old Aire-deficient and control rats (Fig. 4 A). We found a broad downregulation of transcripts, including genes encoding *Ins2*, *Nts*, and *Gip* that have been shown to be differentially expressed in the Aire knockout mouse thymus (Table S1).

Interestingly, our gene ontology analysis for the downregulated genes showed enrichment of pathways associated with “humoral immune response” and “defense response to virus” (Fig. 4 B). Indeed, in addition to TRAs, we found an overt downregulation of ISGs in mTECs of young Aire-deficient rats (Fig. 4 A). The finding was unexpected, especially as Aire-deficient rats at the age of 1–2 mo do not show anti-IFNα autoantibodies. To confirm that this is restricted to the thymus and not present in peripheral tissues, we studied the ISG expression in the thymus, spleen, and lymph nodes of young and old Aire-deficient rats by quantitative PCR (qPCR) (Fig. 4 C). The expression of ISGs (*Ifi27*, *Ifit3*, *Oas2*, and *Rtce10*) was indeed decreased in the thymus but not in the spleen or lymph nodes in young Aire-deficient rats. In contrast, the older Aire-deficient rats over 7 mo showed broad downregulation of ISGs in the thymus and peripheral immune organs, likely because of the appearance of anti-IFNα autoantibodies.

Next, we compared mTEC transcriptome data between Aire-knockout rats and mice, as the latter species do not develop autoantibodies to IFNα-s. For this, we used the Aire-deficient mice mTEC^hi^ transcriptome data from previous studies that reported similar approaches for mTEC^hi^ purification and genome-wide gene expression analysis (GSE14365, GSE33878, GSE2585, and GSE85) (Anderson et al., 2002; Derbinski et al., 2005; Giraud et al., 2012; Hubert et al., 2009). After combining the differentially expressed genes from the rat model and four mouse studies, the principal component analysis of Aire-sufficient mouse and rat datasets revealed distinct Aire-dependent gene expression profiles, potentially due to species-specific functional requirements of Aire in the thymus (Fig. 4 D). However, Aire-deficient mTEC^hi^ populations from both rats and mice showed closely related transcriptomes, suggesting that the absence of Aire leads to loss of species-specific transcriptional regulation, converging mTEC transcriptomes to a similar state. To investigate these species-specific differences, we performed hierarchical clustering of Aire-regulated genes in rats and mice. We identified four distinct gene modules, which exhibited either conserved (module 2) or species-specific (modules 1, 3, and 4) effects of Aire (Fig. 4 E). Notably, module 2, which contained Aire-regulated genes in both species, was enriched in TRAs (odds ratio [OR] = 2.25, P < 1e-05) such as *Ins2*, *Mup1*, and *Gip*. The result indicated that Aire’s role in regulating the expression of TRAs in mTECs is conserved across the two species. Interestingly, cluster 2 was also enriched in ISGs (OR = 4.31, P < 0.01) such as *Oas2*, *Mx1*, *Mx2*, *Ifit2*, *Ifit3*, *Ifitm1*, *and Ifi205*. We then studied the Aire-deficient rat and mouse datasets specifically for the expression of ISGs. To avoid confounding by differences in statistical power between different studies, we performed gene set enrichment analysis (GSEA) on ISGs. GSEA showed downregulation of ISGs in both mouse and rat datasets (P < 0.05). The extent of downregulation, as measured by the normalized enrichment score, varied among datasets. However, the rat dataset showed stronger downregulation than three out of four mouse transcriptome results (Fig. 4, F and H). Nevertheless, when we studied the expression of ISGs in the whole thymus of Aire-deficient mice by qPCR, we found downregulation of 4 out of 13 studied ISGs, indicating that Aire-deficient mice also have decreased expression of ISGs on whole thymus level (Fig. 4 I).

These results show that in addition to TRAs, Aire promotes the expression of ISGs in the thymus, with more prominent ISGs change in rat mTECs than in mice. They also show that impaired thymic T1 IFN signaling in early life precedes the development of anti-IFNα autoantibodies, as it may also be in human patients.

### Suppression of IFN-induced signaling in peripheral immune tissues

**Figure 5.**
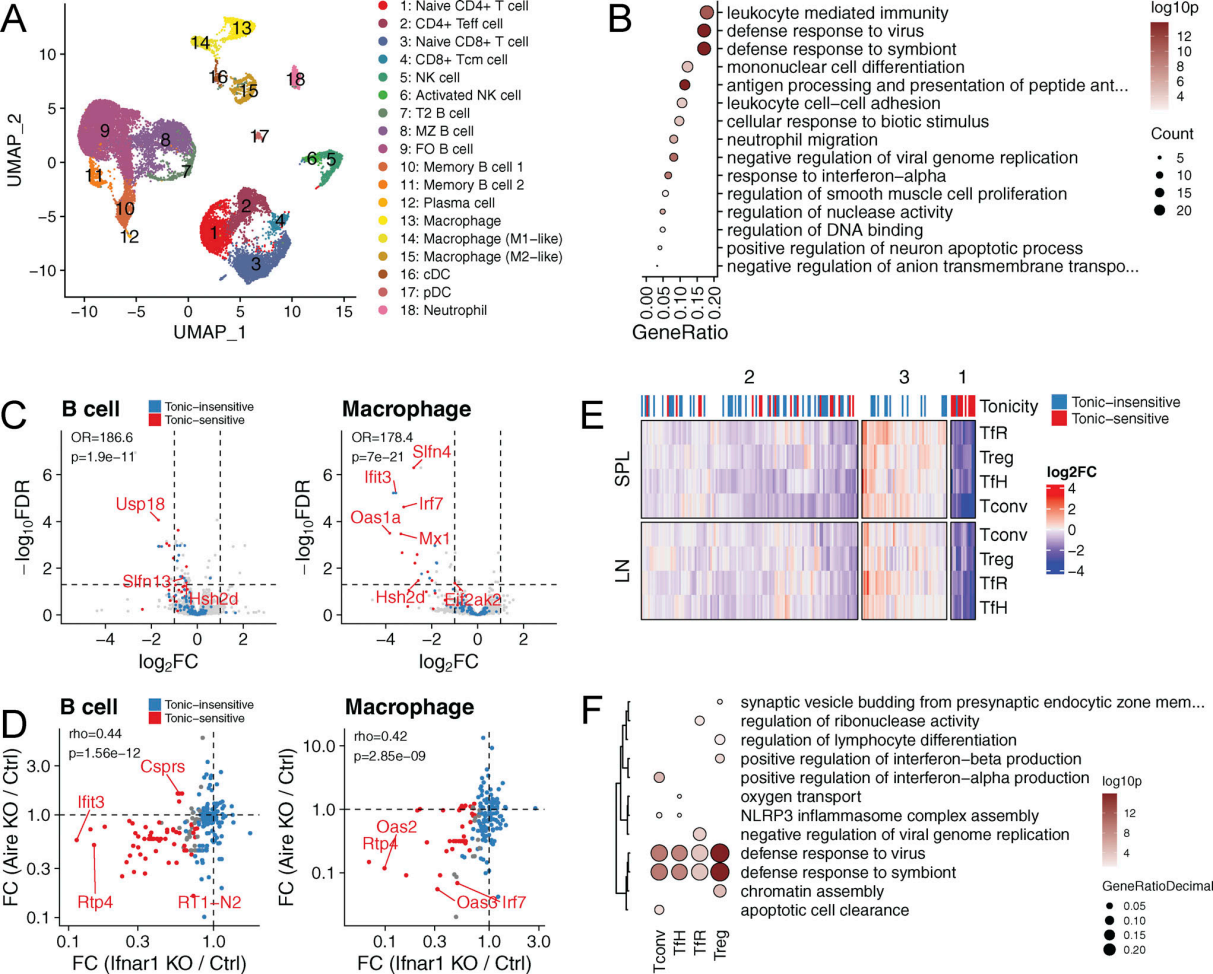
**Diminished IFN signature in splenic immune cells. (A)** UMAP embedding of scRNA-seq data colored by cell type. **(B)** Gene ontology (GO) enrichment analysis of genes differentially expressed across all cell types. Only 15 statistically most significant GO terms are shown. **(C)** Volcano plots showing changes in expression of tonic-sensitive (red) and tonic-insensitive (blue) ISGs in B cells and macrophages. Fisher’s exact test was performed to quantity enrichment of tonic-sensitive genes among downregulated (LFC > 1, FDR < 0.05) genes. **(D)** Scatterplots comparing relative gene expression of tonic-sensitive (red) and tonic-insensitive (blue) ISGs in B cells (left) and macrophages (right) of Aire-deficient and Ifnar1-deficient mice. Spearman correlation coefficient R and associated P value are shown in both figures. **(E)** Heatmap showing relative expression of ISGs in CD4^+^ T cell subpopulations: follicular regulatory (TfR), regulatory (Treg), follicular helper (TfH), and conventional (Tconv) of Aire-deficient rats as compared with control rats. The bar on the right indicates tonic sensitivity of an ISG (SPL, spleen; LN; lymph node). **(F)** GO enrichment analysis of differentially expressed genes CD4^+^ T cell subpopulations in Aire-deficient rats. Data in A and B are obtained from a single experiment with three Aire-deficient (KO) and three control (HE) rats per group. Data in E and F are obtained from a single experiment with 4 Aire-deficient (KO) and 4 control (HE) rats per group. LFC, log fold change.

**Figure S4.**
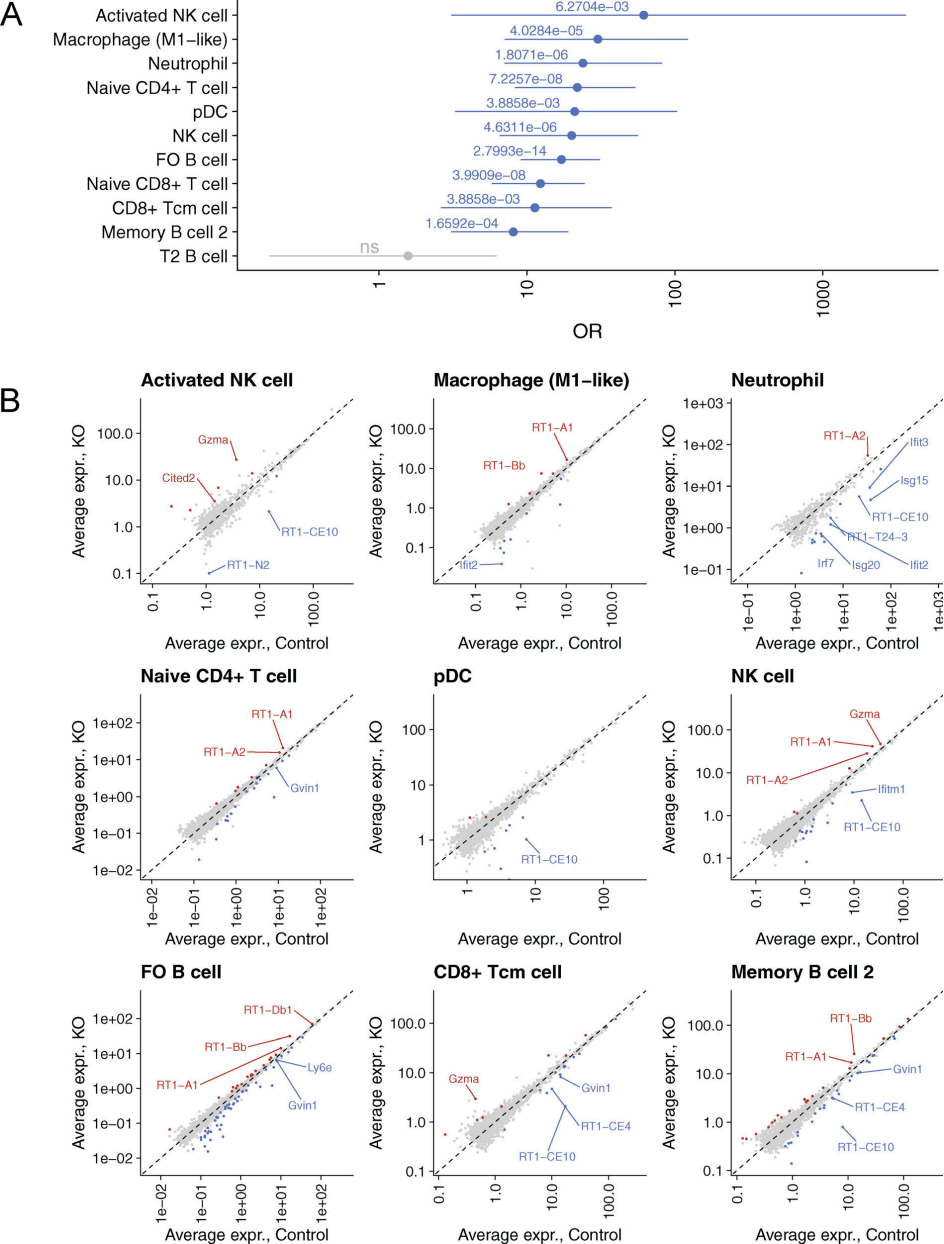
**ISG expression in splenic cell populations. (A)** OR representing enrichment of ISGs among downregulated (FDR < 0.05, LFC less than −1) genes. Activated NK cells show the highest downregulation of ISGs among all splenic cell populations. P values (Fisher’s exact test) are shown, and populations with significant downregulation of ISGs (P < 0.05) are shown in blue. P values are adjusted using Holm–Bonferroni method. ns (not significant) for P ≥ 0.05. **(B)** Average ISG expression in cell populations with the most significant ISG downregulation, based on A. Upregulated and downregulated genes are shown in red and blue, respectively; ISGs are labeled. Data are obtained from one experiment with four Aire-deficient (KO) and three control (HE) animals per group.

Next, we analyzed whether the development of anti-IFNα autoantibodies in older Aire rats resulted in changes in immune cell populations or gene expression patterns in the peripheral immune tissues. We used scRNA-seq to characterize the CD45^+^ splenic cells from 7-mo-old Aire-knockout rats with high levels of anti-IFNα autoantibodies versus age-matched control animals. Using known marker genes, we annotated the CD45^+^ splenic cells into 18 cell clusters (Fig. 5 A and Fig. S3 B), in which we did not see significant differences in main cell populations between the genotypes (Fig. S3 C). The gene ontology enrichment analysis of differentially expressed genes pooled across all cell populations showed “defense response to the virus” and leukocyte-mediated immunity” as the top pathways (Fig. 5 B). The downregulation of ISGs in the rats with high anti-IFNα autoantibodies was prominent in the majority of spleen cells with sufficient gene expression data and the strongest odd ratio effect in activated NK cells and macrophages (Fig. S4, A and B).

We then used a published dataset of B cells and macrophages from the Ifnar1-knockout mouse (Mostafavi et al., 2016) to parse the transcriptional network of T1 IFNs in Aire-deficient rat splenocytes. We separately examined ISGs responsive to induced (acute signal) or tonic (i.e., continuously expressed without an acute IFN signal) levels of IFNs. We found that tonic-sensitive ISGs were preferentially downregulated in B cells and macrophages of Aire-deficient rats and their expression correlated with the ISGs from Ifnar1-knockout mice (Fig. 5, C and D). This showed that the IFNα autoantibodies, associated with Aire deficiency, decrease the basal expression of ISGs in the splenic cell populations.

To further support this finding in splenic cells, we used flow cytometry to isolate four key T cell populations (conventional T cells [Tconv], Treg, T follicular helper cells [Tfh], and T follicular regulatory cells [Tfr]) involved in immune regulation from spleens and lymph nodes and undertook gene expression profiling. Similar to the scRNA-seq results, bulk transcriptome analysis showed downregulation of ISGs in all four T cell subtypes, with tonic-sensitive ISGs showing the most significant downregulation (Fig. 5 E and Table S2). Further, gene ontology enrichment analysis of downregulated genes revealed a strong T1 IFN signal associated with “defense response to virus” (Fig. 5 F). Thus, the anti-IFNα autoantibodies strongly affect the expression profiles of peripheral immune cells and suggest that they have a role in regulating peripheral immune responses in rats and humans.

### Aged Aire-deficient animals have impaired splenic NK cell activation

**Figure 6.**
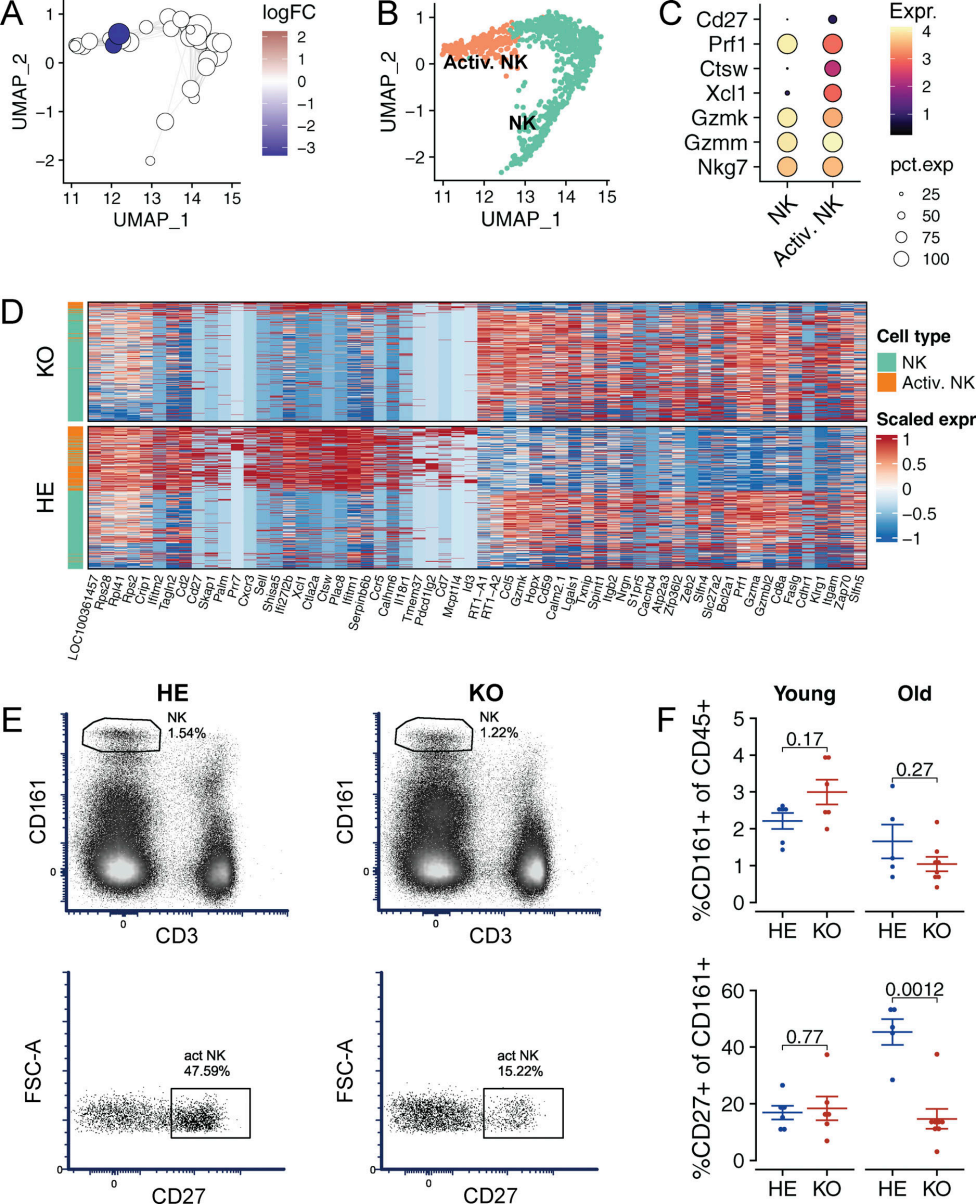
**Compositional and transcriptional changes in NK cells of Aire-deficient rats. (A)** A graph of miloR NK cell neighborhoods superimposed on the UMAP embedding of the data. Color represents log fold change (LFC) estimated with differential neighborhood abundance testing (FDR < 0.05), node size represents the size of the neighborhood, and edge width represents the amount of overlap between neighboring neighborhoods. Empty circles in white represent neighborhoods with statistical significance above 0.05. **(B)** UMAP embedding of NK cells showing a subpopulation of activated NK cells in orange. **(C)** Dot plot showing the expression of activated NK cell marker genes. **(D)** Heatmap showing expression of top 30 marker genes for the two NK subpopulations. Left annotation shows diminished numbers of activated NK cells in Aire-deficient rats. **(E)** Representative flow cytometry plots of splenic NK cells (above) and activated splenic NK cells from >7-mo-old Aire-deficient (KO) and control (HE) rats. **(F)** Mean values of splenic NK cells (above) and activated splenic NK cells (below) from 1–2-mo-old (young) and >7-mo-old (old) Aire-deficient (KO) and control (HE) rats. Data in A–D are obtained from a single experiment with three Aire-deficient (KO) and three control (HE) rats per group. In F symbols indicate individual animals and horizontal lines with whiskers indicate mean value with SEM (*n* = 5–8, combined from two independent experiments). P values are based on *t* tests. P values are adjusted for multiple comparisons using the Holm–Bonferroni method.

Although the main splenic cell populations in the Aire-deficient rats with high anti-IFNα autoantibody levels were comparable in numbers to those in controls, we found a decrease in the activated NK cell subset. To characterize this cell population in more detail, we analyzed compositional changes in the scRNA-seq data using the MiloR package, which identifies differential abundance in overlapping neighborhoods of cells rather than discrete cell clusters (Dann et al., 2022). MiloR tool identified 717 neighborhoods spanning the k-nearest neighbor graph of which three showed evidence of differential abundance (false discovery rate [FDR] < 0.05; Fig. 6 A). All three neighborhoods mapped to the cluster containing activated NK cells (Fig. 6 B) expressing genes related to NK cell effector function such as *Cd27*, *Ctsw*, and *Xcl1* (Fig. 6 C), suggesting impaired activation. Additionally, activated NK cells in Aire-deficient rats expressed ISGs, *Ifi27lb*, *Ifitm1*, and *Ifitm2* (Fig. 6 D), consistent with the role of T1 IFN signaling in NK cell activation (Sun and Lanier, 2011). To confirm these scRNA-seq results, we performed flow cytometry on spleens from young (pre-autoantibody) and old (post-autoantibody) Aire-deficient and control animals (Fig. 6 E). In agreement with the scRNA-seq results, the total NK cell proportions remained unchanged, but old Aire-deficient rats had significantly fewer CD27^+^ activated NK cells (Fig. 6 F). This change was not present in young Aire-deficient rats, suggesting that the development of anti-IFNα autoantibodies in older knockout animals inhibits the activation potential of NK cells, indicating a functional consequence of disrupted T1 IFN signaling in the peripheral immune system.

### Anti-IFNα autoantibodies correlate negatively with autoimmune infiltrations in peripheral tissues

**Figure S5.**
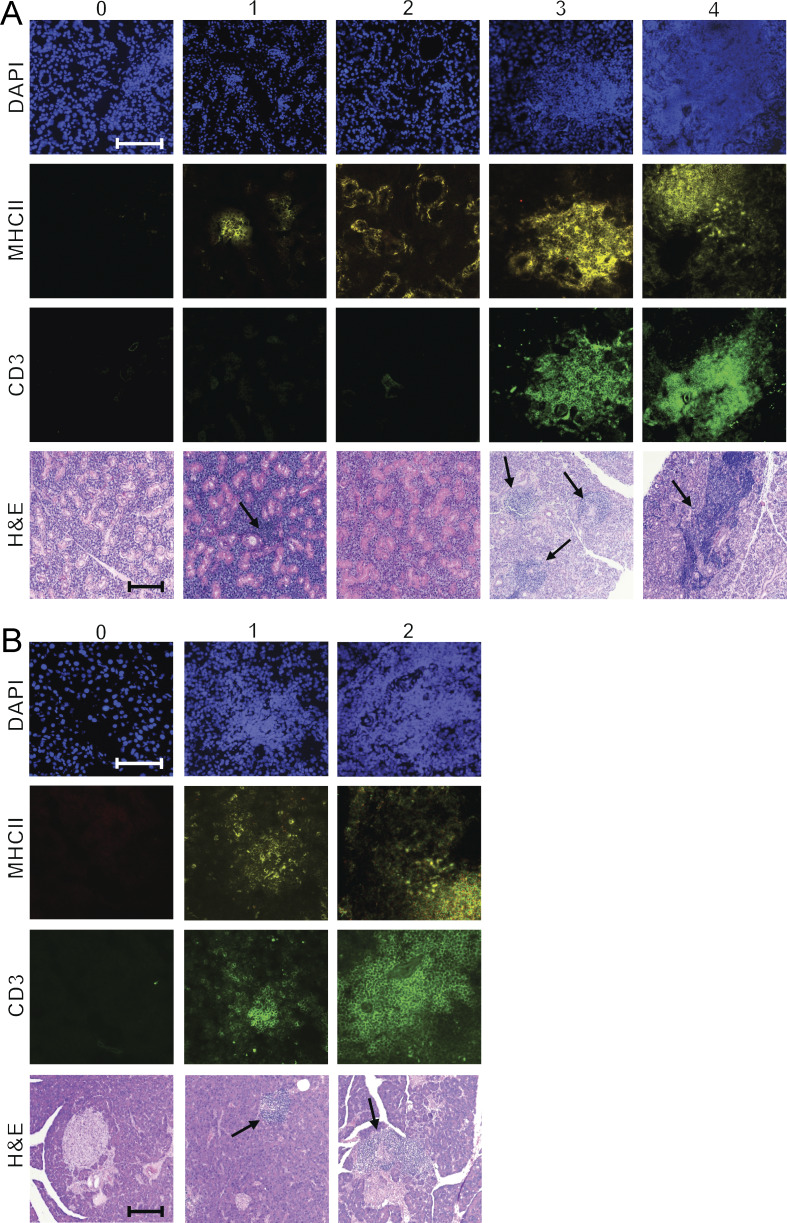
**Pathology score calculation for the salivary gland and pancreas. (A)** Salivary gland: severity of the pathology is graded from 0 to 4. **(B)** Pancreas: pathology is graded from 0 to 2. Scoring is based on immunofluorescence staining (DAPI, CD3, and MHC II), with representative images captured at 200× magnification and a 150-μm scale bar. Corresponding H&E-stained sections at 100× magnification also depict pathology, with black arrows indicating areas of infiltration. Note that some of the H&E stainings are duplicated in Fig. 7.

**Figure 7.**
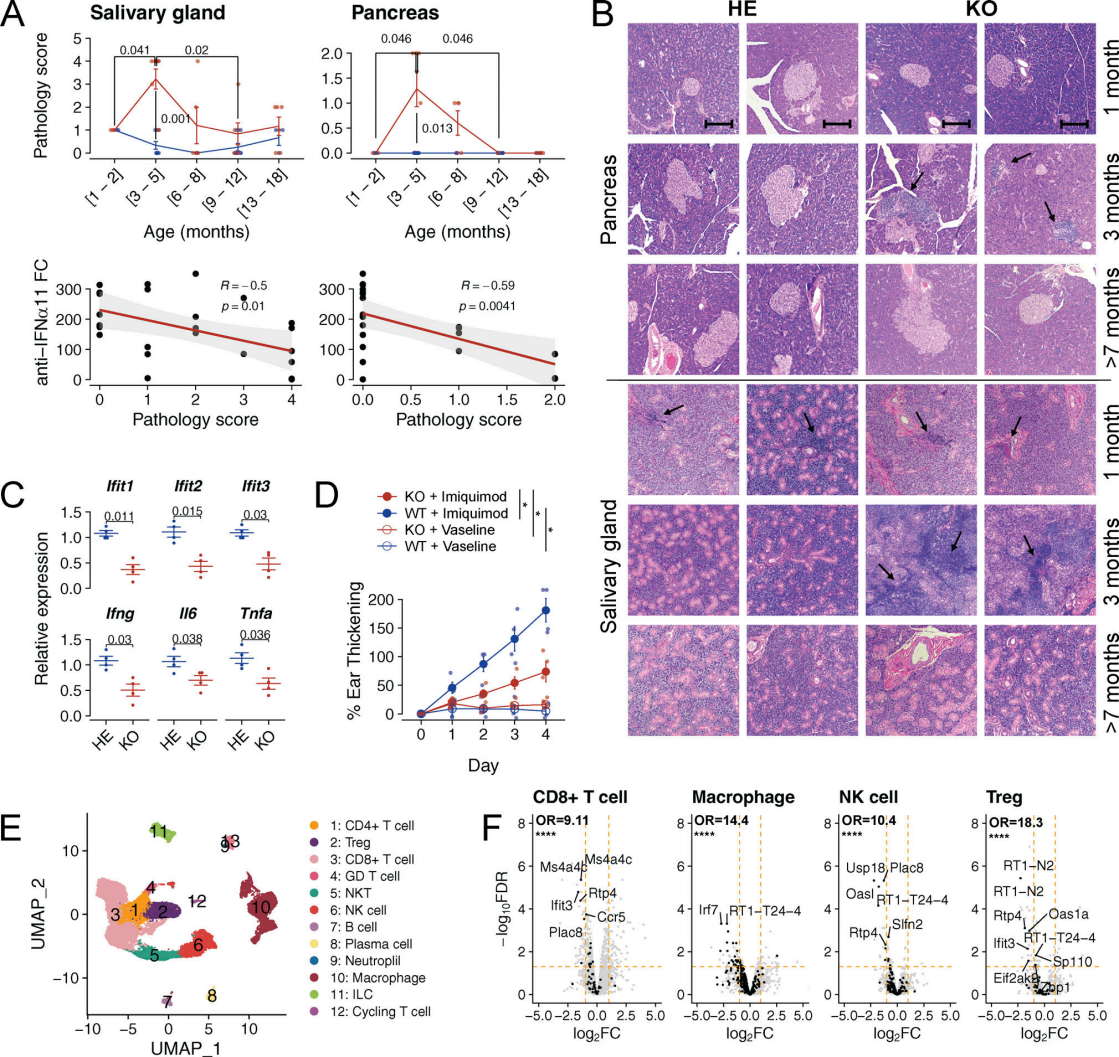
**Increase in anti-IFNα autoantibodies results in a decline in tissue infiltrations and inflammation. (A)** Pathology scores of the salivary gland and pancreas of the Aire-deficient (KO, red) and control (HE, blue) rats across various ages (above) and correlation between anti-IFNα11 and pathology scores of all Aire-deficient animals with detectable autoantibodies (below). Pathology scores were calculated as described in Materials and methods (*n* = 4–8 for each age group and 25 and 22 KO animals for all pooled antibody-positive salivary gland and pancreas samples, respectively; data are combined from three or more individual experiments). P values are based on *t* tests (salivary gland) and the Fisher–Pitman permutation test (pancreas). P values are adjusted for multiple comparisons using the Holm–Bonferroni method. **(B)** Representative samples of H&E (HE) stained pancreatic and salivary gland sections of Aire-deficient (KO) and control (HE) rats (*n* = 4–7 for both groups of KO and HE rats, data are combined from three or more individual experiments). Black arrows highlight cellular infiltrations (100× magnification, scale bar 250 μm). Note that some of the H&E stainings are duplicated in Fig. S5. **(C)** Relative change in expression of ISGs after incubating cultured splenic cells with blood plasma from Aire-deficient rats as quantified using qPCR (*n* = 4 for both KO and HE rats, combined from two independent experiments). P values are based on *t* tests. P values are adjusted for multiple comparisons using the Holm–Bonferroni method. **(D)** The effect of Imiquimod versus vehicle (vaseline) on ear thickening in Aire-deficient (KO) and control (WT) rats determined as described in the Materials and methods (shown as mean value with SEM, *n* = 4 for both KO and WT rats, data obtained from a single experiment). P values are based on two-way ANOVA with post-hoc Wilcoxon signed-rank test for pairwise comparisons; *P < 0.05. **(E)** UMAP embedding of scRNA-seq data obtained from FACS-sorted CD45^+^ cells purified from the salivary glands of 7-mo-old Aire-deficient (KO) and control (HE) rats and colored by cell type. **(F)** Volcano plots showing changes in the expression of ISGs (red) in CD8^+^ T cells, macrophages, NK cells, and Treg cells from the salivary glands of 7-mo-old Aire-deficient and control rats. Data in E and F are obtained from a single experiment with four Aire-deficient (KO) and three control (HE) rats per group. In A and C, symbols indicate individual animals and horizontal lines with whiskers indicate mean with SEM. Fisher’s exact test was performed to quantify enrichment of ISGs among downregulated (LFC < −1, FDR < 0.05) genes. Statistical significance is indicated as follows: ****P < 1e-4.

Some of the most commonly targeted tissues in Aire-deficient mouse models include the salivary gland and pancreas, which exhibit lymphocytic infiltrations and autoantibody reactivity (Kuroda et al., 2005; Niki et al., 2006). Applying pathology scores for the salivary gland and pancreas, calculated based on immunofluorescence staining for CD3 and MHC II markers, we studied the Aire-deficient and control rats over a range of 1–18 mo (Fig. S5). The pathology scores peaked in Aire-deficient rats at 3–6 mo of age, but surprisingly, they decreased gradually thereafter as the rats grew older, becoming indistinguishable from controls at an older age (Fig. 7 A). The same pattern was evident in the salivary glands and the pancreas stained with hematoxylin–eosin where we could not detect mononuclear cell infiltrations in Aire-deficient rats older than 7 mo (Fig. 7 B). Notably, the decrease in the salivary gland and pancreas pathologies was inversely related to the development of anti-IFNα autoantibodies (Fig. 7 A), suggesting a protective effect of the autoantibodies in reducing the autoimmune disease pathology.

We next wanted to demonstrate the effect of the autoantibodies on the IFN signaling pathway using TLR agonists in both in vitro and in vivo settings. The blood plasma from the old Aire-deficient animals suppressed ISG expression in splenocytes stimulated by the TLR3 agonist poly(I:C), suggesting an impaired inflammatory response as a result of high antibody titers in the aged animals (Fig. 7 C). To further confirm that the anti-IFNα autoantibodies have a functional effect in vivo, we applied TLR7 agonist imiquimod to induce the IFN pathway-dependent inflammation in old Aire-deficient and control rats. The imiquimod treatment resulted in dramatic thickening of the ears of control rats, which was significantly reduced in animals with the mutated Aire gene (Fig. 7 D).

To investigate the impact of anti-IFNα autoantibodies on immune cell infiltrations, we sorted CD45^+^ cells from the salivary glands of 7-mo-old Aire-deficient rats and controls and analyzed them by scRNA-seq. Graph-based clustering revealed 13 cell populations, including CD4^+^ and CD8^+^ T cells, Tregs, γδT cells, NK, and NKT cells (Fig. 7 E). Among these, CD8^+^ T cells and macrophages were the main populations among the tissue-infiltrating cells in both Aire-deficient rats and controls. Interestingly, compared with controls, three out of four Aire-deficient animals studied had a smaller proportion of CD8^+^ T cells in their salivary glands (Fig. S3, D and E). Pseudobulk differential expression analysis of the main infiltrating cell populations, CD8^+^ T cells, macrophages, NK cells, and Tregs showed decreased expression of several ISGs, including *Ifit3*, *Ifi47*, *Irf3*, and *Isg20* (Fig. 7 F). This suppression of ISG expression further supports the role of anti-IFNα autoantibodies in mitigating IFN signaling and reducing immune cell infiltration. Taken together, our findings show that the development of T1 IFN autoantibodies in Aire-deficiency is strongly associated with the suppression of IFN signaling and diminished immune cell infiltrations in peripheral tissues.

## Discussion

Here, we characterize the anti-IFNα autoantibodies and their dynamics over time and identify autoantibody-producing cells in the immune tissues of the Aire-deficient rat model. The anti-IFNα autoantibodies start to emerge after 2 mo of age, and all animals are positive for the autoantibodies by 12 mo. They broadly neutralize and affect many immune cells, including T and B cell subsets, in the spleen and lymph nodes, but with the most significant impact being on activated NK cells. The downregulation of ISGs in the thymus occurs prior to the development of anti-IFNα autoantibodies, suggesting a critical role of Aire in establishing immune tolerance to IFNα. Notably, we found the increasing levels of the anti-IFNα autoantibodies to be associated with the age-related decreases in peripheral tissue pathology, suggesting that their anti-inflammatory effects are ameliorating, as was reported for anti-IFNα autoantibodies in APECED patients (Meyer et al., 2016).

The young Aire-deficient rat thymus showed normal thymocyte development but substantially reduced numbers of thymic Tregs. Also, the thymic epithelial compartment was comparable with control animals, suggesting that the impaired IFN signaling does not affect the mTEC differentiation. Predictably, the sorted mTECs showed downregulation of multiple TRAs, including *Ins2*, *Gip*, and *Nts*, and our comparison with published mouse datasets demonstrated both shared and species-specific Aire target genes. Further, single-cell analysis identified three mTEC subsets expressing Aire in the rat thymus: proliferating mTEC II.1, mTEC II.1, and mTEC II.2, of which the latter presented the highest expression of Aire, Aire-dependent genes, and CD86.

Importantly, we show that Aire controls the tonic IFN signal in the rat thymus at an early age, before the generation of anti-IFNα autoantibodies. Tonic T1 IFN signal has emerged as a modulator of homeostatic balance and T cell differentiation in the thymus (Martinez and Hogquist, 2023). IFNα signals are present in the medullary area of the human thymus (Colantonio et al., 2011; Ragazzini et al., 2023), suggesting that it is an evolutionarily conserved feature of the mammalian thymus. Although the cause for the tonic expression is unclear, increasing evidence suggests that the low-grade expression of T1 IFNs influences the thymus function. Previous studies have implicated T1 IFNs in the survival (Moro et al., 2011) and late-stage differentiation (Xing et al., 2016) of thymocytes and induction of thymic Tregs (Metidji et al., 2015; Hemmers et al., 2019). Recently Ashby et al. (2024) showed a critical role of IFNβ and IFNλ signaling in thymic Treg generation and suggested that Aire-dependent expression of T1 IFNs in mice is needed to provide tolerance to ISG-derived self-antigens. Furthermore, IFNs can upregulate the expression of MHC and costimulatory molecules on antigen-presenting cells (Rhodes et al., 1986; Theofilopoulos et al., 2005), including type 1 conventional dendritic cells (DCs), macrophages, and B cells (Ashby et al., 2024), affecting thymopoiesis. Although we saw Aire deficiency to impair the expression of IFN signals in both species, the more prominent downregulation of IFNs in Aire-deficient rats might be one explanation for why anti-IFNα autoantibodies emerge in Aire-deficient rats and not in mice.

The direct detection of T1 and T3 IFNs at both mRNA and protein levels is challenging, partly because their expression levels are near the detection threshold (Rodero et al., 2017). Previous analyses of the human thymus showed IFNα protein expression in CD68^+^ thymic macrophages (Meager et al., 2006) and plasmacytoid DCs (pDCs) (Colantonio et al., 2011; Gurney et al., 2004), predominantly in the medullary region. In the mouse thymus, IFNα production has been observed in pDCs, with significantly reduced expression in Aire-deficient mice (Wang et al., 2019). Importantly, studies using IFNβ- and IFNλ-reporter mice have revealed Aire-dependent IFNβ and IFNλ expression in 1–2% of the mTEChi population, with no detectable expression in hematopoietic cells (Lienenklaus et al., 2009; Benhammadi et al., 2020; Ashby et al., 2024). Ashby et al. (2024) further demonstrated that impaired IFN sensing in the thymus leads to reduced Treg selection, diminished TCR diversity, and increased autoreactive T cell responses to self-antigens expressed during peripheral IFN signaling. Together, these findings provide compelling evidence that Aire-induced constitutive T1 and T3 IFN expression in mTECs contributes to the establishment of a tolerant thymic environment.

Our results showed that downregulated IFN signal in the thymus preceded autoantibodies. Thus, it is tempting to propose that T1 IFN expression in the thymic medulla contributes to immune tolerance to T1 IFNs. In addition to APECED, the autoantibodies to T1 IFNs are detected in other conditions with thymic defects, such as in patients with thymoma and IPEX, and in several inborn errors affecting thymic integrity or thymocyte differentiation (Cheng and Holland, 2024; Oftedal et al., 2024). They are also detected in around 4% of individuals over 70 years, suggesting older individuals are more susceptible to losing immune tolerance to T1 IFNs (Bastard et al., 2021) and associated with COVID-19–related deaths (Manry et al., 2022). Thus, the disturbance of healthy thymic function, either by lack of AIRE, neoplasms, genetic defects, or aging, interferes with proper immune tolerance to T1 IFNs. At least in APECED and thymoma, the development of cytokine autoantibodies is not limited to IFNs as they develop autoantibodies to other cytokines including IL-17A, IL-17F, IL-22, IL-12, and IL-1A (Kisand et al., 2010; Meager et al., 2006). The mechanism by which impaired IFN signaling in the thymus leads to the production of anti-IFNα autoantibodies remains unclear. The absence of T1 IFN expression in the Aire-deficient thymus may contribute to aberrant IFN-specific Treg differentiation, potentially influenced by the loss of tonic inflammatory signals in the thymus. However, the lack of T1 IFN expression and the thymic environment may not be the sole factor regulating anti-IFNα autoantibody production as peripheral mechanisms could also play a role.

The physiological role of the autoantibodies in APECED pathology merits further studies; however, our data reveal their anti-inflammatory and disease-ameliorating effect. We saw no infiltrations in salivary glands and pancreas of older Aire-deficient animals, and the decrease in pathology score paralleled with the increase of anti-IFNα autoantibodies. The anti-IFNα autoantibodies in older Aire-deficient animals were able to suppress IFN signaling, leading to reduced inflammatory responses. This was evidenced by diminished ISG expression in splenocytes treated with a TLR3 agonist and attenuated inflammation in vivo following imiquimod-induced TLR7 stimulation. T1 IFNs are implicated in the induction of T1D in human patients and rodent models (Ferreira et al., 2014; Kallionpää et al., 2014), and in APECED patients, the anti-IFNα autoantibodies were associated with the protection against T1D development, suggesting their beneficial role in the disease (Meyer et al., 2016). Nevertheless, the role of T1 IFN in autoimmunity is complex and we also note that although the Aire-deficient rats had less inflammation in salivary glands and pancreas, they maintained severe skin inflammation at an older age.

In conclusion, our findings contribute to the understanding of the critical function of Aire in the regulation of immune tolerance to T1 IFNs and highlight the intricate balance between tolerance and autoimmunity in the context of T1 IFN signaling. They also underscore the potential therapeutic value of T1 IFN autoantibodies in autoimmune diseases, particularly those associated with aberrant IFN signaling.

## Materials and methods

### Rats

Aire-deficient Sprague–Dawley rats were generated at the University of Nantes, France (Ossart et al., 2018) and bred and maintained at the animal facility of the Institute of Molecular and Cell Biology, the University of Tartu, Estonia, and the animal facility of CR2TI, University of Nantes, France. At the indicated time points between 1 and 19 mo, the animals were euthanized using isoflurane followed by the collection of blood and tissues. Throughout the study, heterozygous littermate rats were used as controls. Experiments and procedures were performed at the University of Tartu in accordance with the ethics committee of the Estonian Ministry of Rural Affairs (Luba-139) and at the University of Nantes in accordance with the ethics committee of the Ministère de l’enseignement supérieur et de la recherche (APAFIS#30504-2021031815316688 v4).

### Mice

As a mouse model of Aire deficiency, the CNS1-KO mice on a C57BL/6 background were used. The mice were generated at the Laboratory Animal Centre of Tartu University as described (Haljasorg et al., 2015) and were maintained at the Vivarium of the Institute of Biomedicine and Translational Medicine, University of Tartu. Experiments and procedures were performed at the University of Tartu in accordance with the ethics committee of the Estonian Ministry of Rural Affairs (Luba-105). At 1.5 mo of age, the mice were euthanized using cervical dislocation and their thymi were collected for analysis. Littermate WT mice were used as controls.

### Blood plasma preparation

Blood plasma was prepared from fresh blood by heparinization and centrifugation (300 *g* 10 min) and stored at −20°C.

### Luciferase immunoprecipitation system (LIPS) assay

The coding sequences of rat IFNα4 and IFNα11 were cloned into a modified pPK–CMV–F4 fusion vector (PromoCell, Heidelberg) and the NanoLuc gene from pNL1.3CMV vector (Promega) that was cloned into the plasmid instead of Firefly luciferase. HEK 293 cells were transfected with cloned constructs, and secreted Nanoluc-Ag fusion proteins were collected with the tissue culture supernatant 48 h later. Rat plasma dilutions (1:10) were incubated for 2 h at RT and then overnight at +4°C in buffer A (50 mM Tris [pH 7.5], 100 mM NaCl, 5 mM MgCl_2_, 1% Triton X-100) containing 10^6^ luminescence units of IFNα in 96-well MultiScreen filter HTS plates (Millipore). Immune complexes from overnight-incubated samples were captured onto Protein G Agarose beads (Exalpha Biologicals). After 2 h, the plate was washed, substrate (fumarazine; Promega) was added, and luminescence intensity was recorded for 5 s with a VICTOR 35 plate reader (PerkinElmer). Every sample was run in three parallel reactions.

### Neutralization of IFNα-induced ISG-reporter activity

Blood plasmas from Aire-deficient or control rats were incubated with 10,000 U/ml IFNα (Miltenyi Biotec) in DMEM containing 10% FBS, 1% antibiotics, antimycotics, and Zeocin (Invivogen) for 37°C, 5% CO_2_. Next, the plasmas were added to the RAW-Lucia ISG reporter cell line (Invivogen) and incubated overnight at 37°C, 5% CO_2_. The QUANTI-Luc Gold substrate was added and samples were analyzed with VICTOR X Multilabel Plate Reader (PerkinElmer Life Sciences). The percentage of inhibition was calculated based on negative and positive controls.

### Neutralization of poly(I:C)-induced ISG expression stimulation

Splenocytes (4 million cells per ml) were stimulated with RPMI1640 containing poly(I:C) (1 µg/ml; Invivogen) and 2.5% rat serum for 90 min at +37°C and 5% CO_2_. Stimulated cells were collected into the TRIzol and analyzed by qPCR.

### Tissue collection and single-cell suspension preparation

For single-cell suspension, the spleens and axillary lymph nodes were homogenized using glass slides, and the bone marrow was collected from the femur by centrifugation. Splenic homogenates were processed as described below or frozen in FBS (20% DMSO). Erythrocytes in splenic and bone marrow samples were lyzed using the ACK solution (150 mM NH_4_Cl, 10 mM KHCO_3_, 0.1 mM Na_2_EDTA; pH 7.3) for 3 min at room temperature (RT). The thymi were minced and gravity-sedimented several times in RPMI1640 media containing 2% FBS to analyze the thymic stromal cells and thymocytes. The enriched stromal compartment was enzymatically digested in HBSS media containing collagenase 4 (125 U/ml; Gibco) and DNase1 (15 U/ml; Applichem) for 15 min at 37°C and followed by 40 min digestions with collagenase 4, DNase1, and Dispase (0.75 U/ml; Gibco). The supernatant from this stromal cell enrichment process was used for thymocyte analysis. Submandibular salivary glands were minced and enzymatically digested in HBSS media containing collagenase 4, DNase1, and Dispase for 60 min at 37°C. To avoid cell aggregation, samples were incubated for 2 min at room temperature and the upper phase was collected for analysis.

### Cell culture

The thymi, spleens, bone marrow, and lymph nodes were collected from Aire-deficient rats at indicated time points, and single-cell suspensions prepared as above. Five million cells per ml were cultured in RPMI1640 containing 10% FBS and 1% antibiotic-antimycotic solution for 2 wk at 37°C, 5% CO_2_ followed by supernatant collection and LIPS analysis.

### Immunofluorescence

The thymus, submandibular salivary gland, and pancreas tissues were dissected, frozen, and cut into 5-μm sections. Sections were fixed for 10 min with 4% formaldehyde, permeabilized with 0.3% TritonX and 0.5% BSA for 10 min, and blocked with 1% normal goat serum at RT for 10 min. Sections were incubated overnight with the indicated primary antibody at 4°C, washed three times in PBS, and incubated with a respective secondary Ab for 60 min at room temperature. Slides were washed three times with PBS, and nuclei were stained with DAPI (1 μg/ml) for 10 min, washed in PBS, and covered with fluorescent-mounting medium (Dako) and coverslipped. Images were obtained with LSM710 microscope (Zeiss). The antibodies used are shown in Table S3.

### Flow cytometry and FACS

Antibodies and viability markers used to stain thymocyte subpopulations and NK cells are shown in Table S3. The flow cytometry gating strategies are shown in Fig. S2. For transcriptome analysis, thymic stromal cells (see above) were sorted into Epcam^+^ CD45^−^ UEA1^lo^ MHC^lo^ cTEC, Epcam^+^ CD45^−^ UEA1^med^ MHC^med^ mTEC^lo^, and Epcam^+^ CD45^−^ UEA1^hi^ MHC^hi^ mTEC^hi^ fractions. For scRNA-seq analysis, the thymic stromal cells were sorted into the CD45^−^Epcam^+^ population. The cells from the spleens and the salivary glands were sorted into CD45^+^ population. For transcriptome analysis, the cells from the spleens and lymph nodes were sorted into CD3^+^ CD4^+^ CD25^−^ ICOS^−^ Tconv, CD3^+^ CD4^+^ CD25^+^ ICOS^−^ Treg, CD3^+^ CD4^+^ CD25^−^ ICOS^+^ Tfh, and CD3^+^ CD4^+^ CD25^+^ ICOS^+^ Tfr populations. For NK cell analysis, frozen splenic homogenates were thawed and erythrocytes were lyzed following an overnight rest period in RPMI1640-media as described above. Cells were sorted using a MA900 cell sorter (Sony Biotechnology) or analyzed with LSRFortessa flow cytometer (BD Bioscience) or SONY ID7000 spectral cell analyzer (Sony Biotechnology). Cells were sorted into Trizol for transcriptome analyze or into PBS for scRNA-seq analysis. All experiments were analyzed using FCS Express 5 Flow Cytometry Software (De Novo Software).

### Whole transcriptome analysis

RNA was purified from sorted cells of thymi, spleens, or lymph nodes using the RNeasy Micro kit (Qiagen). RNA quality was measured with Agilent RNA 6000 Pico kit (Agilent Technologies) and samples with RNA integrity number over 7.5 were used in the analysis. Transcriptome analysis was performed with Genetitan MC (Affymetrix) machine on Clariom S Pico Assay HT (Thermo Fisher Scientific) gene expression chip according to producer introductions. Data were analyzed with a transcriptome analysis console program (Affymetrix). The transcriptome data from Aire-deficient rats were analyzed using the transcriptome analysis console software and compared with four published transcriptome data of sorted mTECs from Aire-deficient mouse model datasets GSE14365 (sorting CD45^−^, MHCII^hi^, Ly51^lo^), GSE33878 (CD45^−^, CD80^hi^, Ly51^lo^), GSE2585 (CD45^−/lo^, EpCAM^+^, CD80^hi^, Ly51^−^), and GSE85 (CD45^−^ EpCAM/G8.8^+^, CDR1/Ly51^int^ and CD80/B7.1^hi^) by converting mouse genes to rat orthologs. Gene set enrichment analysis (GSEA) was performed by first downsampling all gene sets to the same size and then running the analysis using clusterProfiler R package (4.12.6) with 10,000 permutations.

### Real-time qPCR

To extract RNA from whole thymi and spleens, tissues were homogenized in TRIzol manually using a pestle. Total RNA was then purified using the RNeasy Mini kit (Qiagen). RNA was converted to cDNA using the Superscript III kit (Invitrogen) according to the manufacturer’s instructions. Real-time qPCR was carried out using the ViiA7 Real-Time PCR System (Applied Biosystems).

### scRNA-seq library preparation and analysis

Single cells were captured using the 10x Chromium microfluidics system, and barcoded cDNA libraries were prepared using the single-cell 3′ mRNA kit (10x Genomics). Single-cell libraries were sequenced on Illumina MiSeq platform. The data were mapped to the *Rattus norvegicus* reference genome (Rnor_6.0) and quantified using the Cell Ranger software suite (Version 6.0.1, 10x Genomics, Inc.). The Scrublet algorithm was used to identify doublets that were removed from downstream analysis (Wolock et al., 2019), and the default set of filters was used to exclude cells as outliers, as described previously (Germain et al., 2020). Seurat’s sctransform algorithm was used to normalize and scale the data with default parameters (Hafemeister and Satija, 2019), and individual samples were then harmonized using Seurat’s integration pipeline (Stuart et al., 2019).

Pseudotemporal ordering of cells was inferred using Slingshot, and genes whose expression changed along the trajectory were identified using tradeSeq, which fits a negative binomial generalized additive model to expression data to obtain a smoothed expression estimate and tests the null hypothesis that gene expression is not a function of pseudotime (Van den Berge et al., 2020).

T1 IFN score was calculated as described previously (Nezos et al., 2015). For each cell type, the mean and standard deviation of expression of a set of tonic-sensitive ISGs in control rats were used to standardize expression levels of corresponding genes in each Aire-deficient rat. The standardized expression levels were summed for each cell type in Aire-deficient rats to provide an “IFN score.”

Rat- and mouse-specific TRAs were identified using data as previously described in Sansom et al. (2014). Briefly, samples were first reduced to 11 groups using hierarchical clustering to avoid over-representation of similar tissue types. Tissue groups are ranked according to expression level and tissue-restricted genes are called using the dynamic step method. In this method, a gene is called tissue-restricted if its expression level in a few *j* groups is higher than a threshold value *T*, which is a function of the expression level *E* of that gene in the next highest (*n*-*j*th) group:

$$T=\frac{E^{2}}{50}+E+50$$

### Pathology scores

We used immunofluorescence microscopy to evaluate the severity of inflammation in the salivary glands and pancreas of Aire-deficient rats and their control animals. This approach identified CD3^+^ cells, primarily T cells, and MHC II^+^ cells, mainly encompassing B cells, DCs, and macrophages. Our analysis used a 0–4 phenotypic scoring to evaluate the inflammation in the salivary gland and 0–2 scoring to evaluate the inflammation in the pancreas, shown in Fig. S5: score 0: no expression of either CD3 or MHC II detected; score 1: sparse, small infiltrates of MHC II^+^ CD3^−^ cells around ducts; score 2: absence of infiltrates, but widespread expression of MHC II in the glandular parts of the tissue; score 3: presence of medium-sized infiltrates with distinct zones of MHC II^+^ and CD3^+^ cells, alongside MHC II expression in glandular parts; score 4: large infiltrates with clearly demarcated areas of MHC II^+^ and CD3^+^ cells, with glandular sections also expressing MHC II. For each pathology score, we selected H&E-stained sections that represented the observed inflammatory patterns. The staining involved isolating salivary glands or pancreases, fixing in 4% paraformaldehyde in PBS, paraffin embedding, sectioning at 5-μm intervals, and staining with hematoxylin and eosin. Imaging was conducted using a Nikon Eclipse Ci microscope.

### Imiquimod-induced skin inflammation

Male and female Aire-deficient animals and WT control rats, aged 8 mo, were used for the experiments. Skin inflammation was induced in the right ear of each animal by applying daily 12.5 mg of 5% Imiquimod cream (Aldera; 3M Pharmaceuticals) for four consecutive days. The left ear served as a control and was treated with an equal amount of vaseline. Ear thickness was measured daily using a digimatic caliper before treatment and at the same time each subsequent day. The percentage of increase in ear thickness relative to day 0 (baseline) was calculated for each day. Animals were sacrificed on day 5, 24 h after the final Imiquimod application.

### Online supplemental material

This article includes five supplemental files. Fig. S1 shows the analysis of anti-IFNα autoantibodies in APECED patients and individual Aire-deficient rats. Fig. S2 contains an experimental gating strategy for flow cytometry. Fig. S3 contains figures on scRNA-seq annotations and cell compositions in the Aire-deficient and control rat thymic epithelial cells, splenocyte samples, and CD45^+^ cells from salivary gland infiltrations. Fig. S4 shows the extent of ISG expression in Aire-deficient compared with control rats. Fig. S5 shows the pathology score calculation for the salivary gland and pancreas infiltrations. Table S1 contains mTEC transcriptome data from Aire-deficient and control rat and four mouse studies, and differentially expressed ISGs. Table S2 contains fold change transcriptome data in four T cell subtypes from Aire-deficient and control rats. Table S3 lists antibody clones used in the study.

## Supplementary Material

Table S1 contains mTEC transcriptome data from Aire-deficient and control rat and four mouse studies, and differentially expressed ISGs.

Table S2 contains fold change transcriptome data in four T cell subtypes from Aire-deficient and controls rats.

Table S3 lists antibody clones used in the study.

## Data Availability

Transcriptomic data are openly available in a public repository. The datasets generated and analyzed in this study are publicly available in the Dryad repository. Data for transcriptome profiling of CD4^+^ T cells (https://doi.org/10.5061/dryad.mpg4f4r9z) and medullary thymic epithelial cells (https://doi.org/10.5061/dryad.p8cz8wb1s), as well as scRNA-seq of CD45^+^ cells from the salivary gland (https://doi.org/10.5061/dryad.5qfttdzhj), thymic epithelial cells (https://doi.org/10.5061/dryad.8kprr4xz1), and splenic CD45^+^ cells (https://doi.org/10.5061/dryad.hhmgqnksj) from Aire-deficient rats are available. The rest of the data are in the published article and its online supplemental material or available upon reasonable request to the corresponding authors. The code necessary to reproduce the figures and results presented in this study is available at https://github.com/plezar/Aire-Rat-JEM.
